# Blocking Caspase-1/Gsdmd and Caspase-3/-8/Gsdme pyroptotic pathways rescues silicosis in mice

**DOI:** 10.1371/journal.pgen.1010515

**Published:** 2022-12-02

**Authors:** Lulu Kang, Jinghong Dai, Yufang Wang, Peiliang Shi, Yujie Zou, Jingwen Pei, Yaqiong Tian, Ji Zhang, Visarut Codey Buranasudja, Jingyu Chen, Hourong Cai, Xiang Gao, Zhaoyu Lin

**Affiliations:** 1 State Key Laboratory of Pharmaceutical Biotechnology, MOE Key Laboratory of Model Animals for Disease Study, Jiangsu Key Laboratory of Molecular Medicine, Model Animal Research Center, National Resource Center for Mutant Mice of China, Nanjing Drum Tower Hospital, School of Medicine, Nanjing University, Nanjing, China; 2 Department of Respiratory and Critical Care Medicine, Nanjing Drum Tower Hospital, Nanjing, China; 3 Jiangsu Key Laboratory of Organ Transplantation, Wuxi People’s Hospital, Nanjing Medical University, Wuxi, China; 4 Department of Pharmacology and Physiology, Faculty of Pharmaceutical Sciences, Chulalongkorn University, Bangkok, Thailand; HudsonAlpha Institute for Biotechnology, UNITED STATES

## Abstract

Millions of patients suffer from silicosis, but it remains an uncurable disease due to its unclear pathogenic mechanisms. Though the Nlrp3 inflammasome is involved in silicosis pathogenesis, inhibition of its classic downstream factors, Caspase-1 and Gsdmd, fails to block pyroptosis and cytokine release. To clarify the molecular mechanism of silicosis pathogenesis for new therapy, we examined samples from silicosis patients and genetic mouse models. We discovered an alternative pyroptotic pathway which requires cleavage of Gsdme by Caspases-3/8 in addition to Caspase-1/Gsdmd. Consistently, *Gsdmd*^*-/-*^*Gsdme*^*-/-*^ mice showed markedly attenuated silicosis pathology, and *Gsdmd*^*-/-*^*Gsdme*^*-/-*^ macrophages were resistant to silica-induced pyroptosis. Furthermore, we found that in addition to Caspase 1, Caspase-8 cleaved IL-1β in silicosis, explaining why *Caspase-1*^*-/-*^ mice also suffered from silicosis. Finally, we found that inhibitors of Caspase-1, -3, -8 or an FDA approved drug, dimethyl fumarate, could dramatically alleviate silicosis pathology through blocking cleavage of Gsdmd and Gsdme. This study highlights that Caspase-1/Gsdmd and Caspase-3/8/Gsdme-dependent pyroptosis is essential for the development of silicosis, implicating new potential targets and drug for silicosis treatment.

## Author summary

Inhalation of crystalline silica for prolonged periods leads to silicosis, which remains a prevalent occupational disease among mine workers. Silicosis is featured by lung inflammation, pulmonary fibrosis, nodular lesions and eventual development of lung cancer. Millions of patients suffer from silicosis, but it remains an uncurable disease due to its unclear pathogenic mechanisms. Here, we examined samples from silicosis patients and genetic mouse models. We found that Gsdmd and Gsdme both critical for silicosis. If we treated mice with silica, the mice without these two proteins showed markedly attenuated silicosis pathology. Furthermore, we confirm the upstream proteins which activated Gsdmd and Gsdme are Caspase-1, -3 and -8. Those caspases also important for the maturation of IL-1β and IL-18, which are inflammatory cytokines. Finally, we found that inhibitors of Caspase-1, -3, -8 or an FDA approved drug, dimethyl fumarate, could dramatically alleviate silicosis pathology through blocking cleavage of Gsdmd and Gsdme. Our study clarifies the molecular mechanism of silicosis related pyroptosis, and implicates new potential targets and drug for silicosis treatment.

## Introduction

Inhalation of crystalline silica for prolonged periods leads to silicosis, which remains a prevalent occupational disease among mine workers [1–3]. Silicosis is featured by lung inflammation, pulmonary fibrosis, nodular lesions and eventual development of lung cancer [2,4]. The condition of silicosis is progressive and almost always fatal [5]. Due to unclear molecular mechanism of pathogenesis, there is no cure or effective therapy for silicosis, although it is preventable by reducing personal exposure.

The engulfment of pathogens triggers macrophage death and release of inflammatory mediators. Organic pathogens, such as viruses, bacteria and fungi, were cleared after macrophage ingestion [6]. However, phagocytosed sterile particles, such as silica, cannot be destroyed by macrophages, resulting in a subsequent release into the extracellular microenvironment from dying cells. The repeating cycle of particle ingestion and release induces chronic inflammation and further pathological changes in tissues [7]. This process is the root cause of lung inflammation and pulmonary fibrosis in silicosis.

The Nlrp3 inflammasome is involved in the pathogenesis of multiple diseases caused by particles, such as gout, arthritis and asbestosis [3,8–10]. Silica can also activate Nlrp3 inflammasome, subsequently initiates Caspase-1/Gsdmd-dependent pyroptosis and inflammatory cytokine process, which is required for pulmonary inflammation [1,8,9]. However, depletion of Nlrp3 inflammasome complex failed to block silica-induced pyroptosis [1,11]. In addition, previous studies reported that *Caspase-1*^-/-^ mice exhibited sterile inflammatory response, suggesting an alternative signaling pathway regulating inflammatory cell death [12].

Caspase-3 plays an essential role in apoptosis. Recent studies show that cleavage of Gsdme by Caspase-3 converts apoptosis to pyroptosis [13,14]. Previous studies refer to cell death in experimental silicosis as apoptosis due to the presence of activated Caspase-3/-8/-9 and cleaved PARP [15–17]. Though the form of cell death was later reclassified as pyroptosis, suggesting Caspase-3/Gsdme-dependent pyroptosis may also play an essential role in silicosis.

Since pyroptosis is known to be a key process in silica-induced respiratory inflammation, understanding the cellular mediators of particles-induced pyroptosis is mechanistically and therapeutically relevant. In this study, by using both inhibitors and genetic mouse models, we discovered that silica-induced cell lysis and pulmonary inflammation relied on both Gsdmd and Gsdme. Furthermore, we dissected the upstream activation of caspases and downstream release of cytokines in silica-induced pyroptosis, and found a new treatment for silicosis.

## Results

### Silicosis is associated with the activation of gasdermins and related caspases

**Fig 1:**
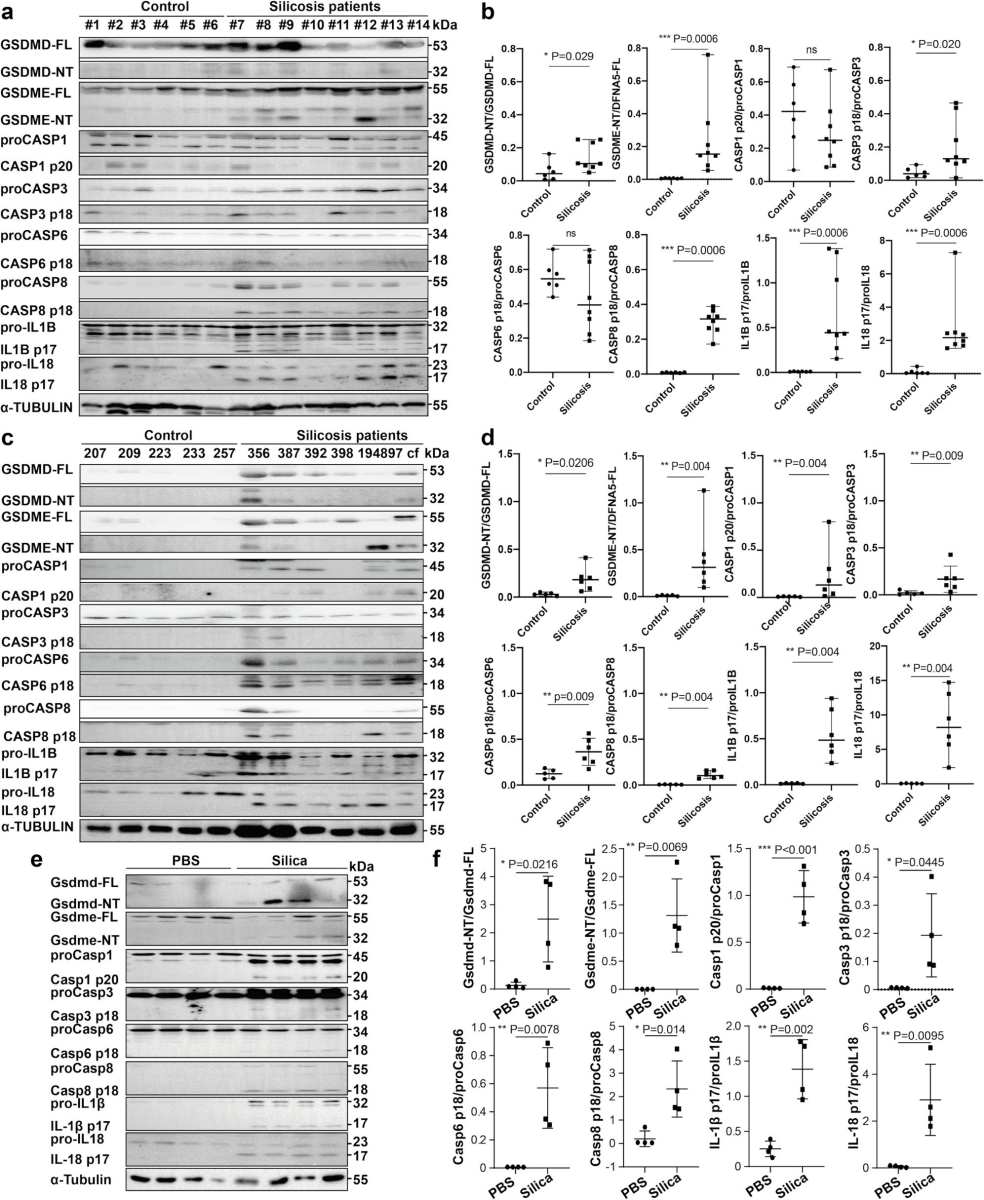
Silicosis is associated with GSDME, GSDMD and caspases cleavage. **a** GSDMD, GSDME, CASP1, CASP3, CASP6, CASP8, IL1B and IL18 cleavage in controls (#1~#6) and silicosis patients (#7~#14) lung tissues. **b** Grayscale analysis of (**a**). **c** Relative protein cleavage in the BALF of controls (207, 209, 223, 233 and 257) and silicosis patients (356, 387, 392, 398, 194897 and cf). **d** Grayscale analysis of (**c**). **e** Gsdmd, Gsdme, Caspase-1, Caspase-3, Caspase-6, Caspase-8, IL-1β and IL-18 cleavage in mouse BALF, *n* = 4. **f** Grayscale analysis of (**e**). Human data were presented as the median ± 95% CI, and analyzed with Mann-Whitney U test. **e** was expressed as mean ± SD. NS, not significant; *P<0.05; **P<0.01; ***P<0.001.

We first checked the activation of pyroptotic pathways in lung tissues from silicosis patients (*n* = 8) and controls (*n* = 6). The results showed that the cleavage of GSDMD and GSDME were significantly enhanced in silicosis patients (Fig 1A and 1B). Furthermore, CASP3, CASP8, IL1B and IL18 were significantly activated (Figs 1A, 1B and S2B). To exclude the influence of non-inflammatory cells, proteins of bronchoalveolar lavage fluid (BALF) collected from silicosis patients (*n* = 6) and controls (*n* = 5) were tested (Figs 1C, 1D and S2B). Consistently, the significantly upregulated activation of GSDMD, GSDME, CASP1, CASP6, CASP3, CASP8, IL1B and IL18 were observed. Thus, these results suggest the potential roles of GSDMD-N and GSDME-N in mediating pyroptosis as occurred in human silicosis.

To confirm the results, the BALF from experimental silicosis mouse models was collected as previously described [8,18]. Consistent with the data obtained in patients, activated Gsdmd, Gsdme, Caspase-1, Caspase-3, Caspase-8, IL-1β and IL-18 were significantly increased (Fig 1E and 1F). In addition, Caspase-6 was significantly activated in mice. Collectively, these data suggested that Gsdmd and Gsdme-dependent pyroptosis were both activated in silicosis.

### Gsdmd and Gsdme are essential for silica-induced pulmonary inflammatory response in mice

**Fig 2:**
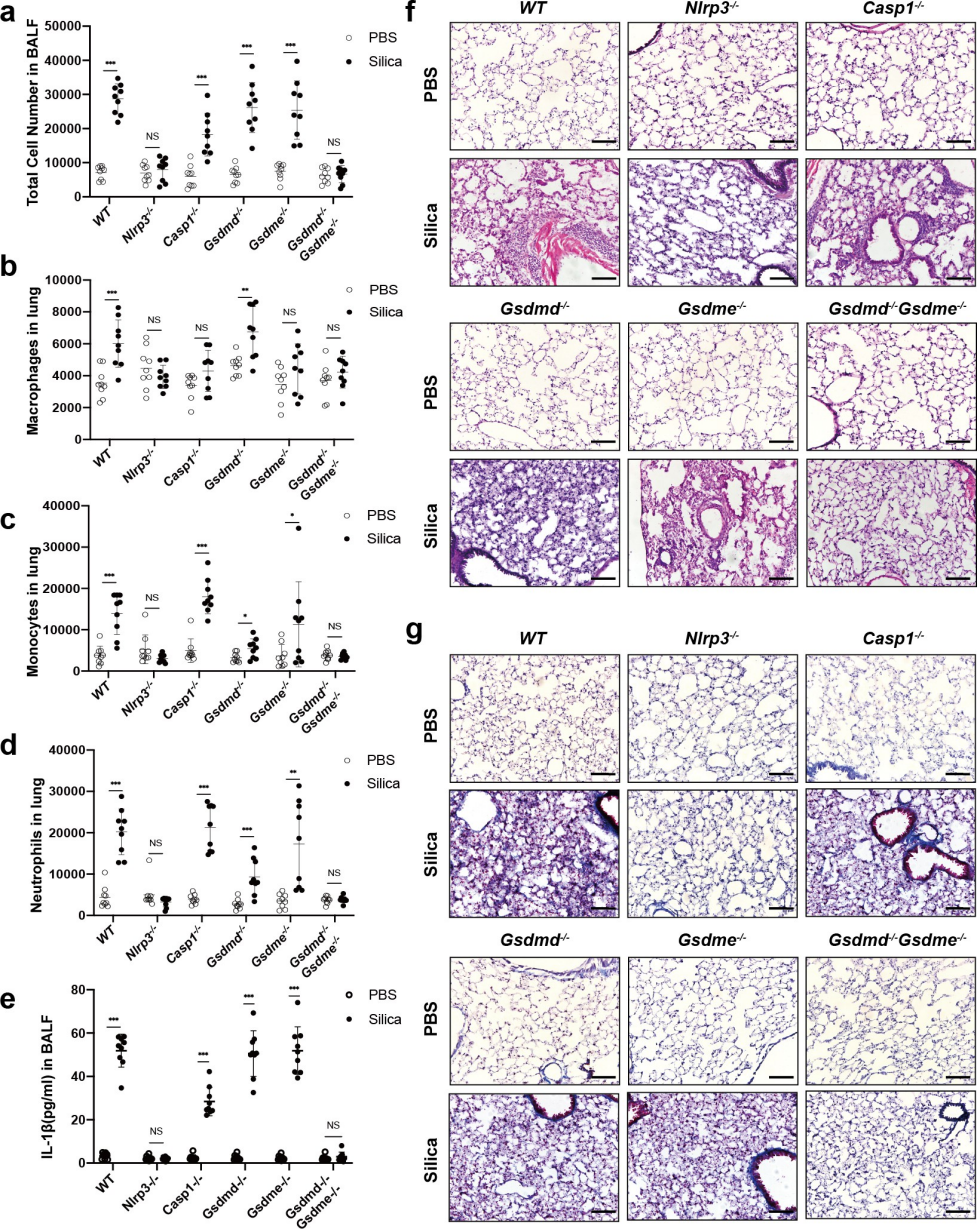
*In vivo* instillation of silica induced pulmonary inflammation alleviates in Gsdmd^-/-^Gsdme^-/-^ mice. **a** The number of cells in the BALF of *WT*, *Nlrp3*^-/-^, *Caspase-1*^-/-^, *Gsdmd*^-/-^, *Gsdme*^-/-^ and *Gsdmd*^-/-^*Gsdme*^-/-^ mice 14 days after exposure to PBS or silica, *n* = 3. **b-d** The number of macrophages (**b**), monocytes (**c**) and neutrophils (**d**) in whole lung tissues, *n* = 3. **e** IL-1β in the BALF of *WT*, *Nlrp3*^-/-^, *Caspase-1*^-/-^, *Gsdmd*^-/-^, *Gsdme*^-/-^ and *Gsdmd*^-/-^*Gsdme*^-/-^ mice 14 days after exposure to PBS or silica, *n* = 3. **f, g** H&E (**f**) and Masson (**g**) staining of mouse lung sections 2 months after the initial challenge. The scale bar represents 100 μm. Results are expressed as mean ± SD from three independent experiments. NS, not significant; *P<0.05, **P<0.01 and ***P<0.001.

To evaluate the physiological relevance of Gsdmd, Gsdme and silicosis, we established experimental silicosis mouse model for *WT*, *Nlrp3*^-/-^, *Caspase-1*^-/-^, *Gsdmd*^-/-^, *Gsdme*^-/-^ and *Gsdmd*^-/-^*Gsdm*e^-/-^ mice. Firstly, we analyzed the immune cells in BALF from mice 14 days after silica exposure. The number of recruiting cells, macrophages, monocytes and neutrophils remained unchanged in *Gsdmd*^-/-^*Gsdme*^-/-^ mice, but were significantly increased in *WT*, *Caspase-1*^-/-^, *Gsdmd*^-/-^ and *Gsdme*^-/-^ mice after silica challenge (Figs 2A–2D and S1B). Consistently, IL-1β and IL-18 secretion in BALF showed similar trends (Figs 2E and S2B), except that IL-18 secretion was not increased in Caspase-1-/- mice. These results indicated that *Gsdmd* and *Gsdme* double deficiency blocked pathogenic immune activation *in vivo*.

**Fig 3:**
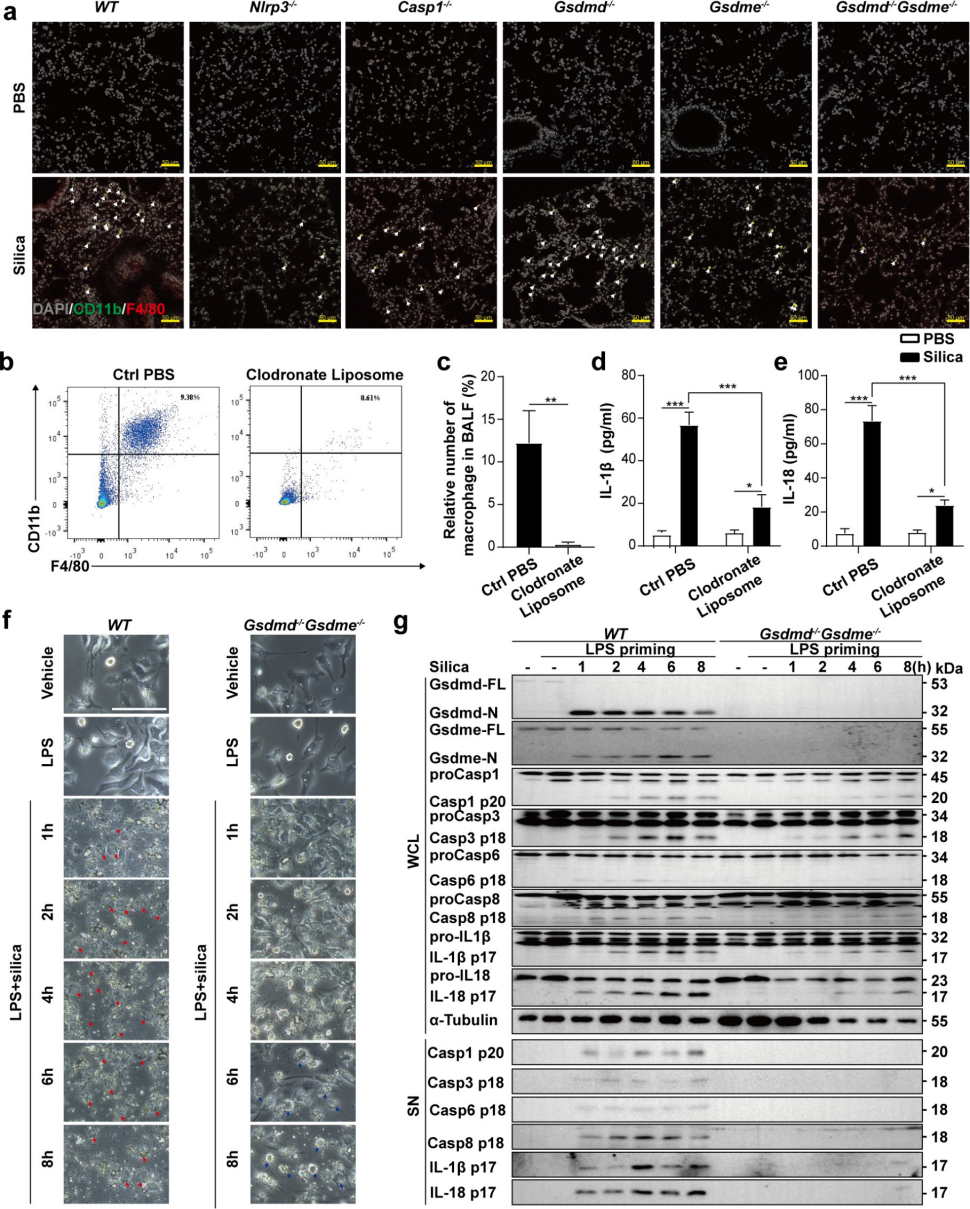
Deficiency of both Gsdme and Gsdmd impairs pyroptosis, proinflammatory cytokine release and immune cell infiltration. **a** Immunofluorescence images of recruiting macrophages in lung tissues 14 days after inhalation of PBS or silica, cells were labelled with antibodies against CD11b (green), F4/80 (red). DAPI (grey) localizes with the nuclei. Arrowheads indicate the infiltrated immune cells. The scale bar represents 50 μm. **b, c** Relative number of alveolar macrophages in BALF of animals, *n* = 3. **d, e** IL-1β (**d**) and IL-18 (**e**) in BALF of mice exposed to silica or PBS, *n* = 3. **f** Images of *WT* and *Gsdmd*^-/-^*Gsdme*^-/-^ macrophages stimulated with silica for 1 h, 2 h, 4 h, 6 h and 8 h. Arrowheads (red) indicate pyroptotic cells, and blue ones indicate apoptotic cells. The scale bar represents 100 μm. **g** Gsdmd, Gsdme, Caspase-1, Caspase-3, Caspase-6, Caspase-8, IL-1β and IL-18 cleavage and secretion in both whole cell lysate (WCL) and supernatant (SN) in *WT* and *Gsdmd*^-/-^*Gsdme*^-/-^ BMDMs treated with silica (0.25 mg/ml). Results are expressed as mean ± SD from three independent experiments. *P<0.05, **P<0.01 and ***P<0.001.

Although acute pulmonary inflammation took place 9~14 days after silica exposure, significant histological changes of lung tissue would be observed 2 months later. After 8 weeks, H&E staining of lung tissue sections showed that there were significant reduction of infiltrating immune cells in both *Nlrp3*^-/-^ and *Gsdmd*^-/-^*Gsdme*^-/-^ mice, as compared to *WT*, *Caspase-1*^-/-^, *Gsdmd*^-/-^ and *Gsdme*^-/-^ mice (Fig 2F). Masson’s trichrome staining showed that *Nlrp3*^-/-^ and *Gsdmd*^-/-^*Gsdme*^-/-^ mice had fewer collagen depositions compared to other groups (Fig 2G). Consistently, the *Nlrp3*^-/-^ and *Gsdmd*^-/-^*Gsdme*^-/-^ mice lung tissues had lower Szapiel scores for H&E staining, suggesting lower inflammation level (S2E Fig). Ashcroft scores for Masson staining also showed less fibrosis level in *Nlrp3*^-/-^ and *Gsdmd*^-/-^*Gsdme*^-/-^ mice lung tissues (S2B Fig). Furthermore, the significantly reduced accumulation of macrophages (CD11b^+^F4/80^+^), monocytes (CD11b^+^Ly6C^+^) and neutrophils (CD11b^+^Ly6G^+^) were observed in lung tissues of *Nlrp3*^-/-^ and *Gsdmd*^-/-^*Gsdme*^-/-^ mice (Figs 3A and S2A). In conclusion, these data demonstrated that double depletion of *Gsdmd* and *Gsdme* attenuates mouse pulmonary inflammation and fibrosis.

### Gsdmd and Gsdme are essential for silica-induced pyroptosis in macrophage

Macrophage is considered as an important cell type in mediating silicosis [1,8]. To determine whether macrophages are the key cells, we specifically depleted macrophages in lung tissues by clodronate liposome *in vivo* (Fig 3B and 3C) [19,20]. BALF from silicosis mice treated with clodronate liposome showed decreased IL-1β and IL-18 production (Fig 3D and 3E). These results demonstrated the pivotal role of macrophages in mediating mouse pulmonary inflammation.

As macrophages play an indispensable role in mediating pulmonary inflammation in silicosis, we explore the underlying molecular mechanisms by using primary bone marrow-derived macrophages (BMDMs) from *WT* and *Gsdmd*^-/-^*Gsdme*^-/-^ mice. After silica treatment, *WT* macrophages started displaying cell membrane rupture at an early time point (1 h), while most *Gsdmd*^-/-^*Gsdme*^-/-^ macrophages maintained cell membrane integrity until 8 h (Fig 3F). The double KO macrophages also showed much less LDH release, and much less Propidium Iodide (PI) positive cells (S2E, S2F and S2G Fig). The caspase activation level was much weaker in *Gsdmd*^-/-^*Gsdme*^-/-^ macrophages than in *WT* macrophages (Fig 3G), suggesting that Gsdmd and Gsdme may be involved in the activation of specific caspases. Consistently, maturation and secretion of IL-1β and IL-18 were also reduced in *Gsdmd*^-/-^*Gsdme*^-/-^ macrophages (Fig 3G). These data demonstrated that double depletion of *Gsdmd* and *Gsdme* reduced cell lysis and cytokine release.

### Caspase-3/-6/-8/Gsdme and Caspase-1/Gsdmd pathways are essential for silica-induced pyroptosis

**Fig 4:**
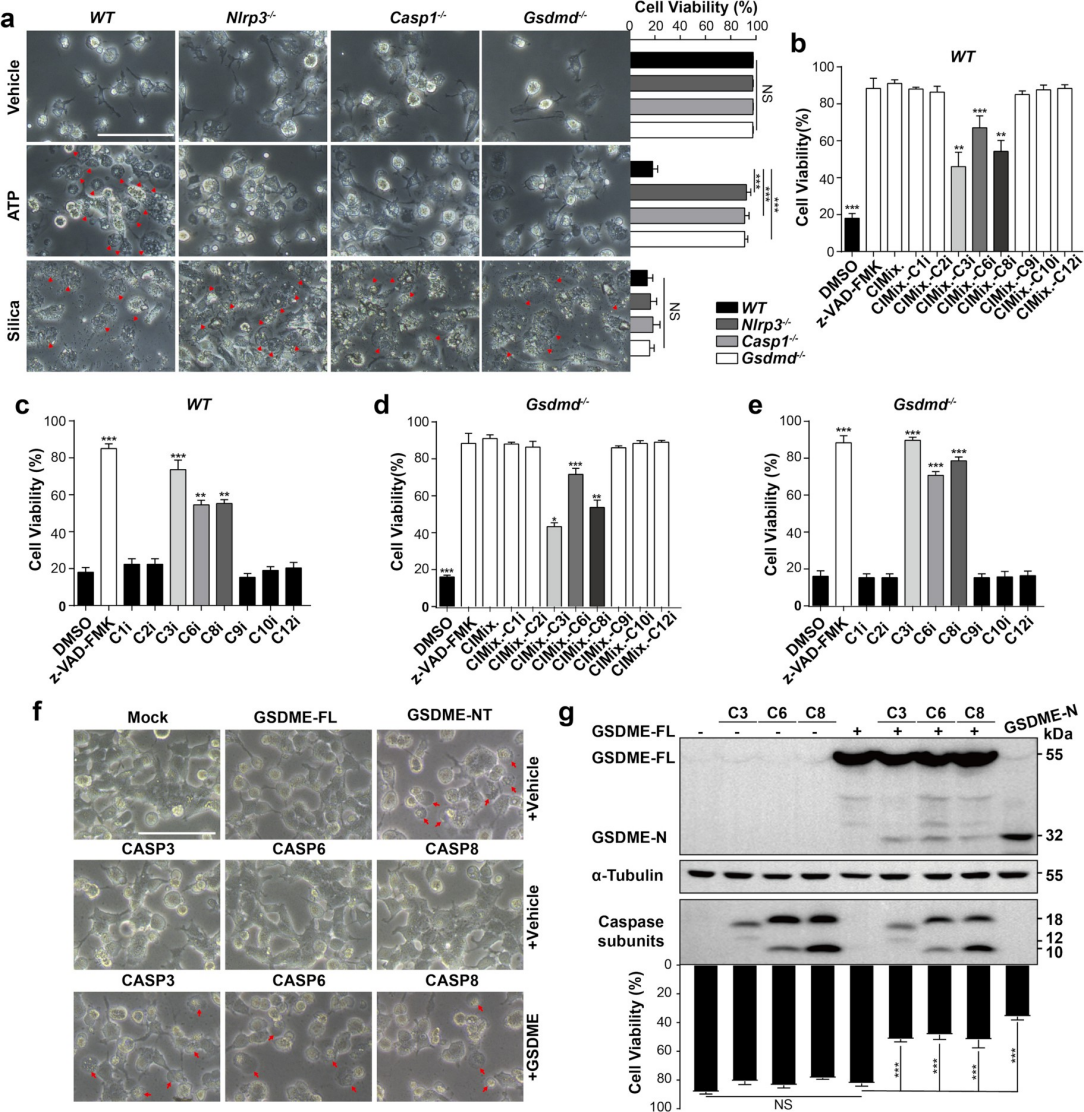
Caspase-3/-6/-8 are required for Gsdme-dependent pyroptosis. **a** Images and cell viability of *WT*, *Nlrp3*^-/-^, *Caspase-1*^-/-^ and *Gsdmd*^-/-^ BMDMs left untreated or stimulated with ATP and silica. Arrowheads indicate pyroptotic cells. The scale bar represents 100 μm. **b** Cell survival of primed *WT* BMDMs pretreated with inhibitor mixture as indicated and stimulated with silica. CIMix represents the mixture of all the indicated caspase inhibitors, while CIMix-C1i means lack of Caspase-1 specific inhibitor and the rest can be deduced by analogy. **c** Cell survival of silica-stimulated *WT* macrophages pretreated with the indicated caspase inhibitors. **d** Cell survival of primed *Gsdmd*^-/-^ BMDMs pretreated with inhibitor mixture as indicated and stimulated with silica. **e** Cell survival of silica-stimulated *Gsdmd*^-/-^ macrophages pretreated with the indicated caspase inhibitors. **f** Images of cells overexpression of GSDME and CASP3, -6, -8 subunits. The scale bar represents 100 μm. a-f The cell viability checked by FACS with PI staining. **g** The cleavage of GSDME and the viability of cells overexpressing GSDME and active CASP3, -6, -8 subunits, the cell viability checked by LDH release. z-VAD-FMK, pan-caspase inhibitor; C1i, VX765 (Caspase-1 inhibitor); C2i, z-VDVAD-FMK (Caspase-2 inhibitor); C3i, z-DEVD-FMK (Caspase-3 inhibitor); C6i, z-VEID-FMK (Caspase-6 inhibitor); C8i, z-IETD-FMK (Caspase-8 inhibitor); C9i, z-LEHD-FMK (Caspase-9 inhibitor); C10i, z-AEVD-FMK (Caspase-10 inhibitor); C12i, z-ATAD-FMK (Caspase-12 inhibitor). The working concentration of the inhibitor mixture was 50 μM. Results are expressed as mean ± SD from three independent experiments. NS, not significant; **P<0.01 and ***P<0.001.

Though silica induces Nlrp3/Caspase-1/Gsdmd-dependent pyroptosis [21,22], it also elicits Nlrp3 inflammasome-independent lytic cell death [1,23]. Our data showed that cleavage of both Gsdmd and Gsdme contributed to pulmonary inflammation (Figs 1 and 2). Furthermore, depletion of either Gsdmd or Gsdme by itself did not protect mice from pulmonary fibrosis (Fig 2). Consistent with previous reports and our data, depletion of *Nlrp3*, *Caspase-1* or *Gsdmd* cannot block lytic cell death in response to silica in BMDMs, while cell lysis induced by ATP was completely Nlrp3/Caspase-1/Gsdmd dependent (Fig 4A). These data confirmed that silica could trigger cell death through an Nlrp3/Caspase-1/Gsdmd-independent manner.

To identify the possible caspases involved in the silica induced cell death in BMDMs, we used mixtures of caspase inhibitors (including inhibitors from Caspase-1 to Caspase-12) that either included the complete list of inhibitors (CIMix) or missed a specific inhibitor. Specific depletion of the Caspase-3, Caspase-6 or Caspase-8 inhibitor resulted in significantly increased cell death (Fig 4B). In addition, the inhibitor of Caspase-3, Caspase-6 or Caspase-8 increased silica-stimulated *WT* BMDMs survival independently (Fig 4C). Similar results were obtained in *Gsdmd*^-/-^, *Nlrp3*^-/-^ and *Caspase-1*^-/-^ macrophages (Figs 4D and S3A–S3D), suggesting that Caspase-3, -6, and -8 related pyroptosis is Nlrp3/Caspase-1/Gsdmd independent. Time lapse PI staining showed that the loss of macrophage membrane integrity was rescued by the Caspase-3 inhibitor (S3E Fig). Furthermore, co-expression of GSDME and CASP3/CASP6/CASP8 separately resulted in lytic cell death (Fig 4F). Cleavage of GSDME and ballooning bubbles in the cell membrane were also observed (Fig 4F and 4FG). Taken together, we demonstrated that Caspase-3/-6/-8/Gsdme-dependent pyroptosis is also essential for cell death.

Silica triggered cell lysis in the absence of either *Gsdmd* or *Gsdme* (S4A Fig). Macrophage deficiency of both gasdermins resulted in resistance to silica-induced pyroptosis (Figs 3F, 3G and S4A). *Nlrp3/Casp1/Gsdmd*-deficient cells treated with Caspase-3 inhibitor and *Gsdme*-deficient cells treated with Caspase-1 inhibitor (VX765) showed similar cell viability to the WT cells treated with pan-caspase inhibitor (S4A Fig), suggesting that Nlrp3/Caspase-1/Gsdmd and Caspase-3/Gsdme pathways both triggers pyroptosis. Furthermore, in Gsdme deficient cells, caspase-3 inhibitor cannot block the cell death, suggesting that caspase-3 is not required for Gsdmd dependent cell death (S4A Fig). In Gsdmd deficient cells, caspase-1 inhibitor cannot block the cell death, suggesting that caspase-1 is not required for Gsdme dependent cell death (S4A Fig). The release of cytokine IL-1β and IL-18 was reduced and was correlated to the inhibition of cell lysis (S4B Fig).

To confirm the relationships between Caspases and Gasdermins, we collected proteins from whole cell lysate and the supernatant of primed macrophages treated with ATP or silica pre-incubated with different caspase inhibitors. As shown, Gsdmd and Gsdme cleavage and related caspase activation were assessed in silica-treated *WT* macrophages, and the inhibitors exhibited strong inhibitory effects (S4C Fig). Cleavage of Gsdme and activation of Caspase-3/-6/-8 were observed in the absence of Nlrp3/Caspase-1/Gsdmd pathway, confirming that Caspase-3/-6/-8/Gsdme is an alternative pyroptotic pathway in silica-induced pyroptosis (S4C Fig).

In addition to inhibiting Caspase-3, z-DEVD-FMK also partially inhibited the activation of Caspase-1, -6 and -8 (S4C Fig). Therefore, to circumvent the side effects of inhibitors, we knocked down Caspase-3, Caspase-6 and Caspase-8 in BMDMs with siRNA (S5A Fig). Individual knockdown of Caspase-3, Caspase-6 or Caspase-8 markedly attenuated silica-induced cell death (S5B Fig). The immunoblot results showed that silencing of Caspase-3, Caspase-6 or Caspase-8 in the cells had no inhibition on other Caspases (S5C Fig). The changes of the activation of Gsdmd, Gsdme, Caspase-1, -3, -6, -8, IL-1β, IL-18 were consistent with the results obtained with the inhibitors (S5C Fig). Collectively, we demonstrated that both Caspase-3/-6/-8/Gsdme and Caspase-1/Gsdmd pathways participate in silica-induced pyroptosis genetically.

### Caspase-1/Gsdmd and Caspase-8/Gsdme both contribute to IL-1β processing and release through an inflammasome-dependent manner

**Fig 5:**
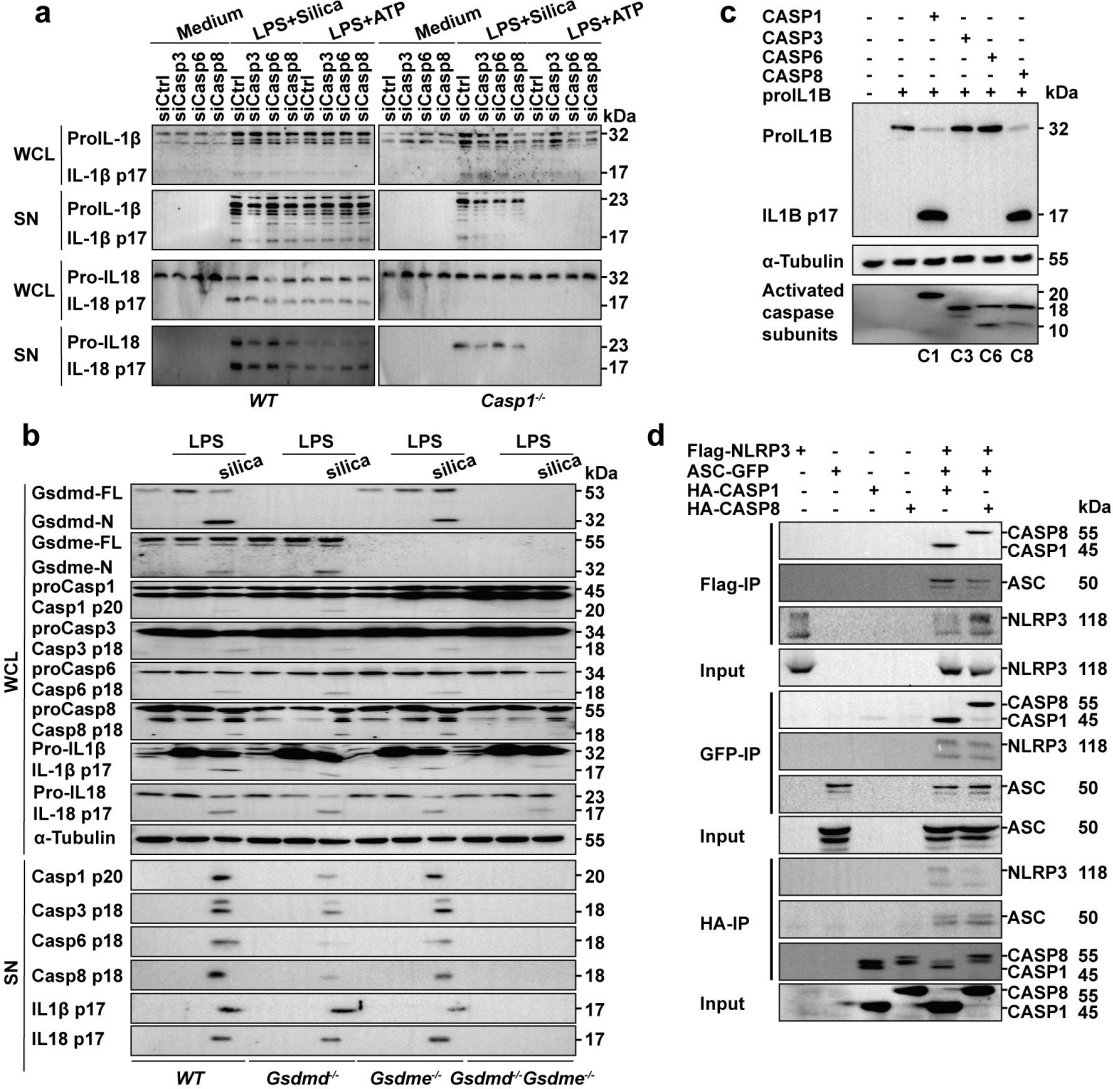
Caspase-1 and Caspase-8 are both engaged in IL-1β maturation and secretion in silicosis. **a** Immunoblot analysis of mature IL-1β and IL-18 in whole cell lysate and supernatant of *WT* and *Caspase-1*^-/-^ BMDMs. **b** Immunoblot analysis of Caspase-1, Caspase-3, Caspase-6, Caspase-8 IL-1β and IL-18 activation and release in whole cell lysate and supernatant of *WT*, *Gsdmd*^-/-^, *Gsdme*^-/-^ and *Gsdmd*^-/-^*Gsdme*^-/-^ BMDMs treated with silica for 2 h. **c** Recombinant proIL1B is directly cleaved by CASP1 and CASP8. **d** Co-immunoprecipitation analysis of the interaction of NLRP3, ASC and CASP1 or CASP8.

IL-1 receptor blockade alleviates pulmonary inflammation and halt the progressive decline in lung function, indicating that IL-1 cytokines are the key factors of pulmonary fibrosis [1,3,12]. The pores formed by Gsdmd-N and Gsdme-N domains provide a rapid way for cytokine secretion [21,24,25]. The released IL-18 stimulated with ATP or silica was blocked by inhibition of the Nlrp3/Caspase-1 pathway (S4B and S4C Fig). ProIL-18 cannot be cleaved when Caspase-1 is deficient, suggesting that the maturation of IL-18 is dependent on Caspase-1 (Fig 5A). However, the release of mature IL-18 was only blocked in *Gsdmd*^-/-^ *Gsdme*^-/-^ macrophages, but not in *Gsdmd*^-/-^ or *Gsdme*^-/-^ cells (Fig 5B). Taken together, maturation of IL-18 is mediated by Caspase-1, but its rapid release is gasdermin-dependent. As previously shown, the deficiency of Nlrp3/Caspase-1/Gsdmd pathway cannot block silicosis, suggesting that IL-1β has a more fundamental role in silicosis.

Conventionally, IL-1β is synthesized as an inactive precursor and remains inactive until converted to an active cytokine after processing by Caspase-1. But our results showed that *Caspase-1* deficiency only reduced, but not fully blocked the cleavage and release of IL-1β both *in vivo* and *in vitro* (Figs 2E and S4B). These results suggested that the maturation and release of IL-1β can be Caspase-1-independent. Through an *in vitro* assay, we found the activation and secretion of IL-1β was reduced in *Caspase-1*-deficient cells while Caspase-8 was silenced (Fig 5A). We also demonstrated that overexpressed activated forms of Caspase-1 and Caspase-8 cleaved proIL1B in 293T cells. (Fig 5C). It suggested Caspase-8 is also involved in the maturation of IL-1β.

Similar to Caspase-1, Caspase-8 can be recruited by the adaptor protein ASC [26,27]. Our previous results showed that Nlrp3 deficiency impaired both Caspase-1 and Caspase-8 activation, accompanied with reduced IL-1β maturation, suggesting that Caspase-8 may be activated by Nlrp3 inflammasome (S4C and S5C Figs). The results of co-immunoprecipitation assay showed that Nlrp3, ASC and Caspase-8 physically interact (Fig 5D). It suggests that, in addition to cleaved Gsdme, Caspase-8 mediated IL-1β maturation in an Nlrp3 inflammasome-dependent manner in silica-induced pyroptosis.

Suppression of Caspase-1 activity not only maintained *Gsdme*^*-/-*^ macrophage survival, but also blocked its secretion of IL-1β (S4C Fig). The release of mature IL-1β was blocked by deficiency of both Gsdmd and Gsdme, suggesting that the rapid cytokine release required pores formed by gasdermins (Fig 5B). Furthermore, our data showed that, in *Gsdmd*^-/-^*Gsdme*^-/-^ BMDMs, maturation of IL-1β was alleviated in cytoplasm, along with the decreased activity of Caspase-1, 3, 6 and 8 (Fig 5B). It is known that activated Gsdmd and Gsdme reversely function on the upstream of specific caspases to initiate cell death [25,45]. Similar mechanism may also exist in silica induced cell death, which explains that deficiency of both Gsdmd and Gsdme decreases the activation level of upstream caspases.

Taken together, these data demonstrated that Caspase-1/Gsdmd and Caspase-8/Gsdme both mediated IL-1β maturation and secretion in silicosis.

### Inhibiting Caspase-3/-6/-8/Gsdme and Caspase-1/Gsdmd pathways reduces silicosis pathology *in vivo*

**Fig 6:**
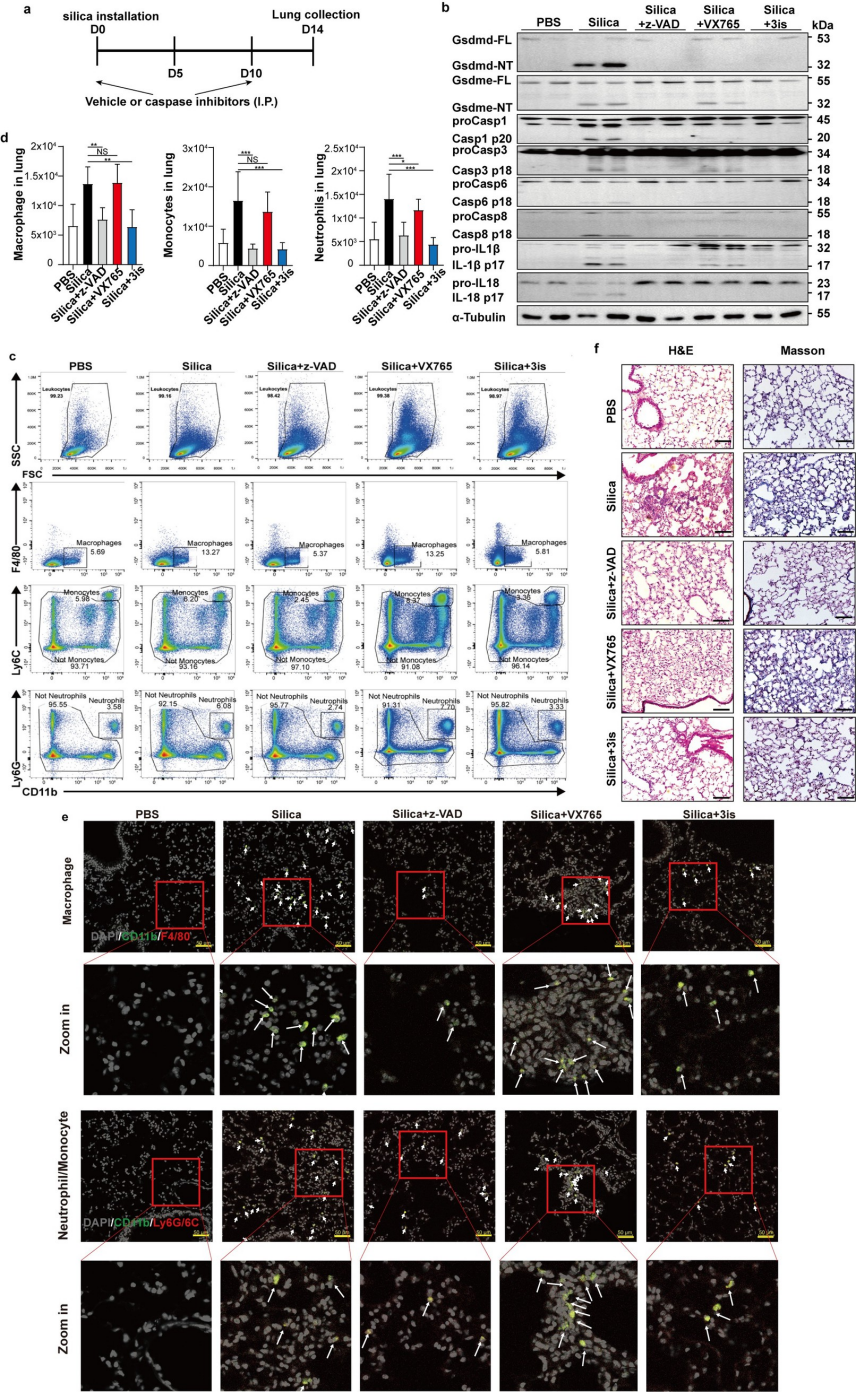
*In vivo* administration of caspase inhibitors upon silica stimulation reduces immune cell infiltration and silicosis pathology. **a,** Caspase inhibitors intervene strategy. **b,** Western blot of Gsdmd, Gsdme, Caspase-1, Caspase-3, Caspase-6, Caspase-8, IL-1β and IL-18 activation in collected BALF from mice treated as indicated, *n* = 2. **c,** Relative number of macrophages, monocytes and neutrophils in the whole lungs of mice 14 days after instillation of PBS, silica and the indicated inhibitors, *n* = 5. **d,** The number of macrophages, monocytes and neutrophils in lung tissue of mice that installed with PBS, silica and indicated inhibitors, *n* = 5. **e,** Immunofluorescence images of macrophages (CD11b^+^F480^+^), neutrophils (CD11b^+^Ly6G^+^) and monocytes (CD11b^+^Ly6C^+^) in lung tissues of mice. Arrowheads indicate the infiltrated immune cells that labelled with antibodies against CD11b (green), F4/80 (red) and Ly6G/6C (red). DAPI (grey) localizes with the nuclei. The scale bar represents 50 μm. **f,** H&E (left) and Masson (right) staining of the indicated mouse lung sections 14 days after the initial silica challenge. The scale bar represents 100 μm. z-VAD, z-VAD-FMK; 3is, VX765+z-DEVD-FMK+z-IETD-FMK. z-VAD-FMK, pan-caspase inhibitor; VX765, Caspase-1 inhibitor; z-DEVD-FMK, Caspase-3 inhibitor; z-IETD-FMK, Caspase-8 inhibitor. The total dosage of injected caspase inhibitor(s) was 0.25 mg per mouse administered once. Results are expressed as mean ± SD from three independent experiments. NS, not significant; *P<0.05, **P<0.01 and ***P<0.001.

Related caspases mediated not only the cleavage of Gsdmd and Gsdme, but also the maturation and release of cytokines. Inhibition of these caspases could be one of the ways to treat silicosis. We designed *in vivo* assays to examine the therapeutic effect of caspase inhibitors in silicosis. At first, we treated mice with ntranasally (i.t.) administered 0.1 ml of sterile PBS or 5 mg of silica particles in 0.1 ml of saline at day 0 (Fig 6A). Intraperitoneal injection of solvent or Caspase inhibitors was carried out at Day 0, Day 5 and Day 10. Mice were sacrificed at 14 days after silica or saline instillation (Fig 6A). Intraperitoneal injection of z-VAD-FMK or 3is (Caspase-1/-3/-8 inhibitors) blocked the activation of pyroptosis markers and cytokines (Fig 6B). Consistently, VX765 failed to inhibit Gsdme cleavage and IL-1β release (Fig 6B). The flow cytometry analysis and tissue immunofluorescence demonstrated that the number of infiltrated macrophages, monocytes and neutrophils were reduced after the z-VAD-FMK or 3is treatment (Fig 6C–6E). Administration of VX765 failed to attenuate immune cell recruitment, which is consistent with the results obtained in *Caspase-1*^-/-^ mice (Figs 2A–2D, 3A and 6C–6E). Lung sections showed that injection of the pan-caspase inhibitor or 3is reduced the levels of fibrosis and collagen deposition (Fig 6F). Furthermore, even if we treated mice 3 days after silica inhalation, intraperitoneal injection of z-VAD-FMK or 3is (Day 3, Day 6, Day 9 and Day 12) still showed significant improvement of silicosis (S6 Fig), suggesting that blocking related caspases can also rescue silicosis in the progress of the disease.

**Fig 7:**
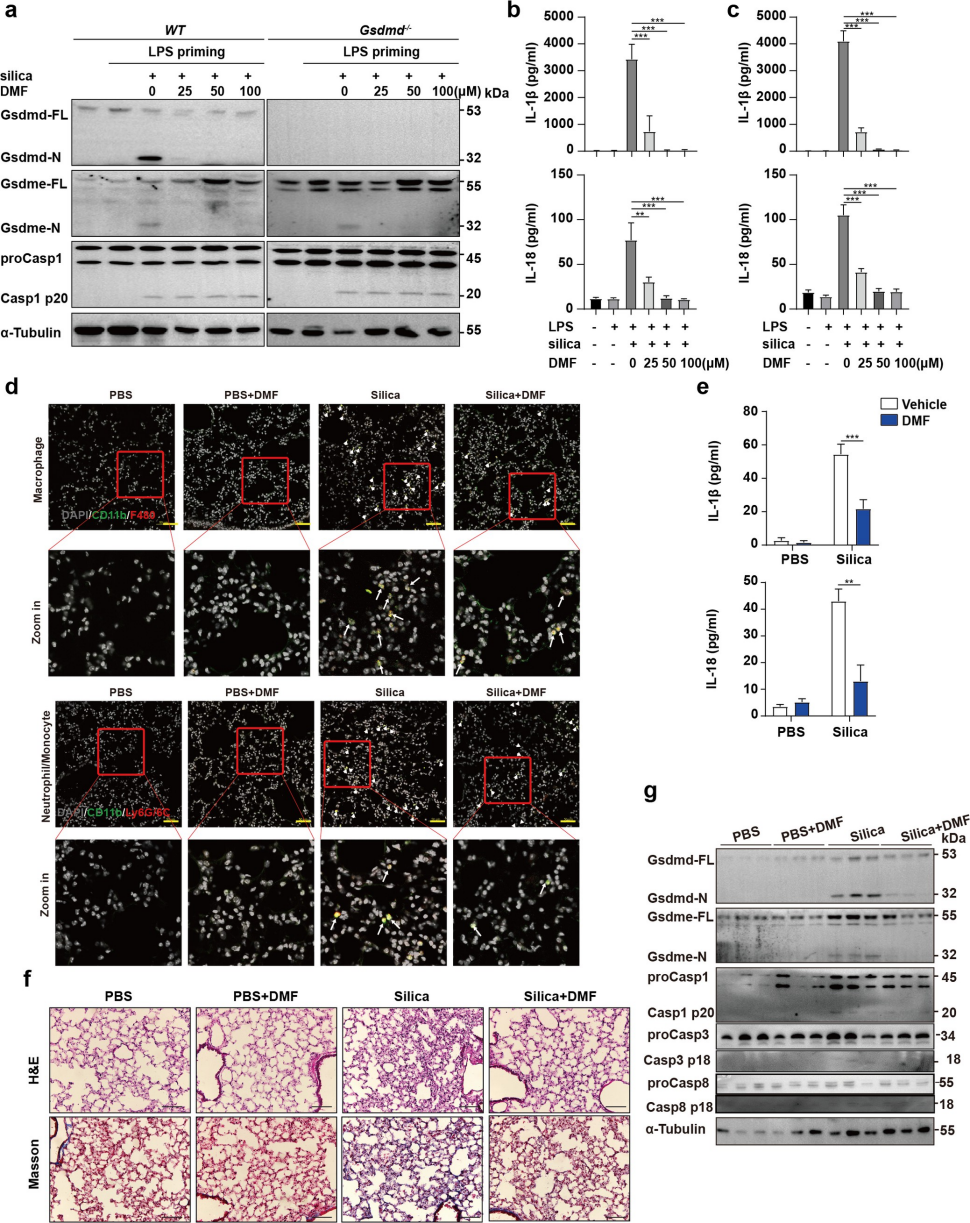
Inactivation of Gsdmd and Gsdme by dimethyl fumarate rescue mouse silicosis. **a** The activation of Gsdmd, Gsdme and Caspase-1 was measured through immunoblot analysis, both in *WT* and *Gsdmd*^-/-^ BMDMs. **b, c** The release of IL-1β and IL-18 in *WT* and *Gsdmd*^-/-^ BMDMs culture medium. **d** Immunofluorescence images of macrophages (CD11b^+^F480^+^), neutrophils (CD11b^+^Ly6G^+^) and monocytes (CD11b^+^Ly6C^+^) in lung tissues of mice. Arrowheads indicate the infiltrated immune cells that labelled with antibodies against CD11b (green), F4/80 (red) and Ly6G/6C (red). DAPI (grey) localizes with the nuclei. The scale bar represents 50 μm. **e** The levels of IL-1β and IL-18 in BALF of mice, *n* = 3. **f** H&E (upper) and Masson (lower) staining of the indicated mouse lung sections 14 days after the initial silica challenge. The scale bar represents 100 μm. **g** The activation of Gsdmd, Gsdme and Caspase-1 was measured through immunoblot analysis. Results are expressed as mean ± SD from three independent experiments. NS, not significant; **P<0.01 and ***P<0.001.

Though the results showed that the combination of caspase inhibitors could alleviate mouse silicosis pathology *in vivo*, application of caspase inhibitors is limited in clinical trials due to their side effects. Succination of Gsdmd and Gsdme by dimethyl fumarate (DMF) prevents their cleavage, oligomerization and capability to induce pyroptosis (Figs 7A, S7A and S7B) [28]. The secretion of IL-1β and IL-18 was also reduced by DMF (Fig 7B and 7C). Thus, we performed *in vivo* experiments to identify the effect of DMF on silicosis. Daily gavage of DMF for 14 days significantly reduced the infiltrated immune cells in lung tissues (Fig 7D), cytokine release (Fig 7E), mouse silicosis pathology (Figs 7F and S7C). The decreased cleavage of Gsdmd and Gsdme by DMF treatment was observed in BALF (Fig 7G).

In conclusion, these results demonstrated that blocking Caspase-1/Gsdmd and Caspase-3/-8/Gsdme-dependent pyroptosis could be the new target of therapy for pulmonary silicosis.

## Discussion

In our study, we found that in addition to the Caspase-1/Gsdmd pathway, Caspase-3/-6/-8 mediated Gsdme cleavage is essential for silica induced pyroptosis. Consistently, a recent study demonstrated that Caspase-3 activation in response to intrinsic and extrinsic apoptotic stimuli is significantly reduced in *Gsdme*-deficient cells when compared with *wild type* cells. Gsdme functions both downstream of Caspase-3 to induce pyroptosis and upstream of Caspase-3 to augment its activation [29]. Egil Lien *et al*. and Alexander Poltorak *et al*. discovered that Caspase-8 activation as a result of inhibition of TAK1 by pathogenic *Yersinia* infection caused cleavage of both Gsdmd and Gsdme in murine macrophages, contributing to pyroptosis [30,31]. In addition, it has been demonstrated that inflammasome drives Gsdmd-independent pyroptosis and Caspase-8-mediated IL-1β release in the absence of Caspase-1 activity [32].

Our results showed that maturation of IL-1β was dependent on inflammasome-activated Caspase-1 and Caspase-8 in silica-induced pyroptosis. In the canonical inflammatory signaling pathway, activated Nlrp3 and the adaptor protein ASC oligomerize through their pyrin domains (PYDs) to form a large multiprotein complex. More than 1,000 proteins are reported to be enriched in this complex platform [32]. It is well known that Caspase-1 is recruited to the inflammasome via the interaction between the caspase recruitment domains (CARD) of proCaspase-1 and ASC. Previous studies have shown that in the CARD-based inflammasome, activation of caspase-8 occurs at the ASC spots during apoptosis of caspase-1-deficient macrophages [26,27]. Activation of Caspase-8 at the inflammasome has been implicated in the non-canonical maturation process of IL-1β [33,34]. Since particles are known to activate the Nlrp3 inflammasome, it has often been assumed that particles cause cell death through Caspase-1/Gsdmd-mediated pyroptosis. Therefore, there exists a compensatory mechanism in which Caspase-8 mediates IL-1β maturation in the absence of Caspase-1 activity, explaining why *Caspase-1* deficiency failed to protect mice from silicosis.

Previous studies have mainly focused on macrophages to explain the molecular mechanism of experimental silicosis. In this study, we also showed macrophages are important for silicosis (Fig 3). However, the role of alveolar epithelial cells has also been studied in many reports [35–37]. Furthermore, neutrophils are reported to be the phagocytic cleaner of particles, similar to the role performed by macrophages [8,38]. Our data also showed that neutrophils are recruited in large numbers to the lungs in silicosis animal models, similar to previous reports (Figs 2D, 6D and S6D) [39–41]. Since the Nlrp3 inflammasome pathway is intact in neutrophils, Gsdmd- and Gsdme-dependent pyroptosis may also occur in neutrophils. In this study, we did not exclude the potential functions of neutrophils and alveolar epithelial cells in silicosis, despite the depletion of macrophages significantly inhibited inflammatory response (Fig 3D and 3E). However, the exact roles of these cells in silicosis need further studies.

There is no increased caspase-1 processing in the human lung tissue from silicosis patients, which is not consistent with human BALF and mice data. Caspase-1 is specific highly expressed in monocytes, macrophage and neutrophils. In the lung tissue, the percentage of these cells is around 5% [42]. Furthermore, the processing caspase-1 only could be detected in dying pyroptotic cells. Since the process of pyroptosis is fast, the percentage of dying pyroptotic cells is low. The low percentage of innate immune cells and the low percentage of dying pyroptotic cells in the tissue may explain that why no significant increased caspase-1 processing was observed in tissues. However, in the BALF, more than 90% are macrophages, the activation of Caspase-1 was obvious [42].

The activation of Caspase-3 and Caspase-6 is not dependent on Nlrp3, suggesting there are other singalings to activate silicosis induced cell death beside Nlrp3 [43,44]. *In vitro* assay showed that the pyroptosis inhibitory effect from Caspase-6 blockage was weaker than that of blocking Caspase-3 or Caspase-8 (Figs 4B–4E and S3A–S3D). These findings suggest that Caspase-6 is not a critical regulator of silicosis, although it is capable of cleaving Gsdme (Fig 4G). Since activation of Caspase-6 is induced by the upstream Caspase-3 in the apoptotic pathway, the Caspase-6 activity we observed in the silicosis models may be a redundant protein function.

Although cleaved Gsdmd and Gsdme reversely activated their related caspases [25,45], the underlying mechanism is still unclear. There are several possible signaling pathways. Gsdmd and Gsdme N-terminus make pores on cell membrane. The ions can pass the pores and activated following inflammasome, like K^+^ efflux and Nlrp3/ASC/Caspase-1 [46]. Furthermore, the released DAMPs from pyroptotic cells could be the triggers for caspases activation [47,48]. Since these pathways may work together to activate caspases, the clarification of detail mechanism needs further study.

DMF is an FDA-approved drug used in transplantation medicine and autoimmune diseases, like multiple sclerosis (MS). Recent reports suggests that Gsdmd dependent pyroptosis play an important role in MS [28,49]. Our data showed DMF also had significant protective effects of silicosis. However, the underlying mechanisms of DMF is still unclear. Blocking cleavage of Gsdmd by DMF provides mechanistic insight into its immunemodulatory activity in MS therapy [28]. Some reports suggest that DMF could inhibit inflammation through IRAK4 and Nrf2 signalling [50]. But considering its affordable cost and effectiveness, DMF may be further recommended to treat silicosis patients in developing countries.

Pyroptosis is a rapid form of cell death. The engulfment of silica by macrophage triggers pyroptosis and acute inflammation. However, silica cannot be destroyed by macrophages, resulting in a subsequent release into the extracellular microenvironment from dying cells. The clearance of silica is relatively slow in vivo. The repeating cycle of particle ingestion and release induces chronic inflammation and further pathological changes in tissues [7]. Furthermore, even after silica was cleared, the fibrosis was hardly reversed. That may be the reason why pyroptosis is important for silicosis, a chronic disease.

**Fig 8:**
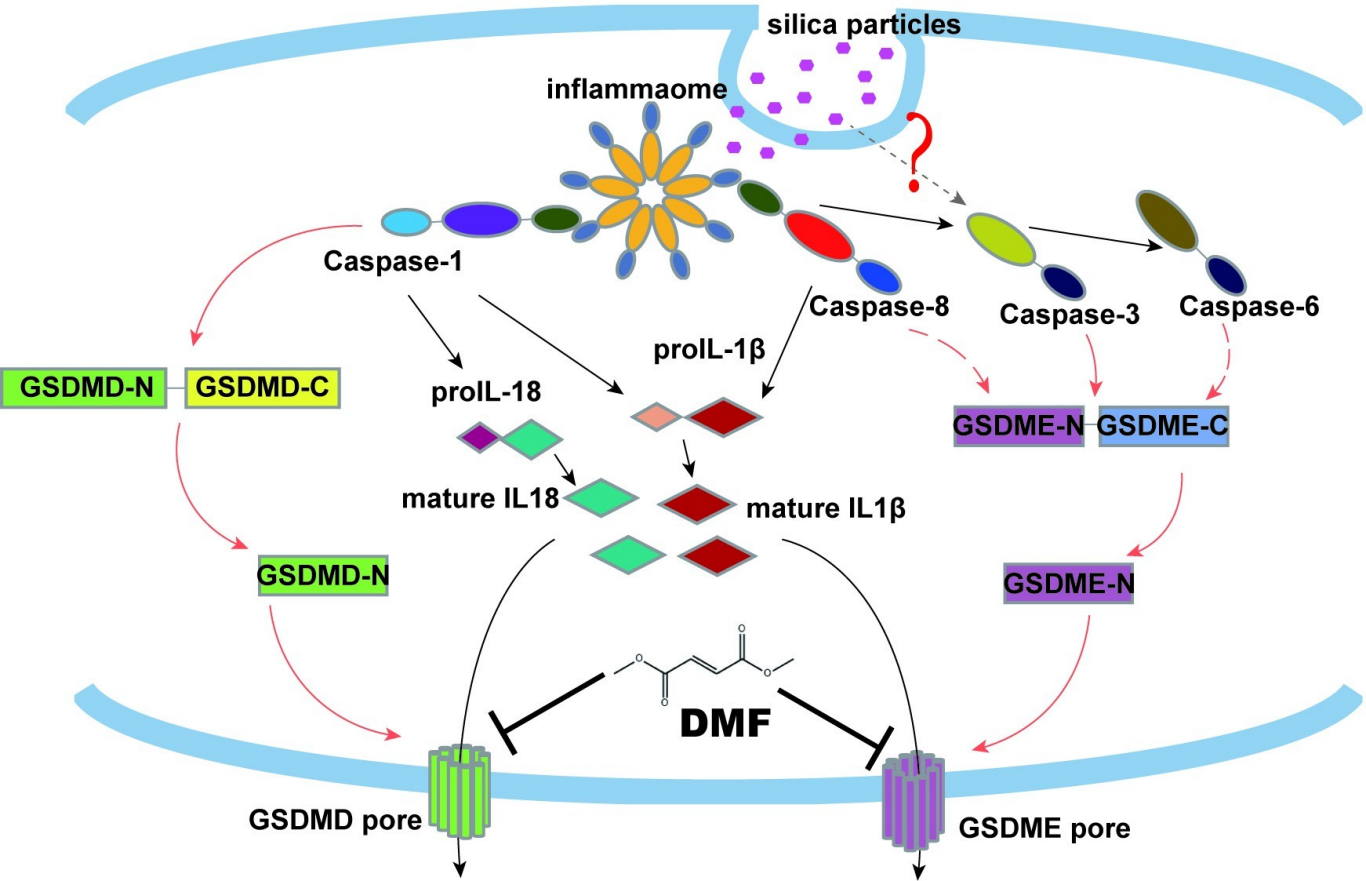
Schematic of silica-induced pyroptosis and inflammation.

Silicosis is an irreversible and progressive fibrotic lung disease, leading to respiratory insufficiency. A better understanding of the molecular mechanism regulating cell death and inflammatory mediator production by macrophages may help identify better therapeutic targets for the disease. We found that Caspase-1/Gsdmd and Caspase-3/-8/Gsdme pathways are both essential for the development of pulmonary inflammation and fibrosis. Inactivation of Gsdme and Gsdmd cleavage significantly blocked silica-induced pyroptosis and alleviated mice pulmonary inflammation and fibrosis (Fig 8). Our findings provide new targets for therapy of silicosis.

## Materials and methods

### Ethics statement

All animal welfare and experimental procedures were approved by the Animal Care and Use Committee of the Model Animal Research Center, Nanjing University (Nanjing, China).

Lung tissue and BALF samples were procured by the Nanjing Drum Tower Hospital, the Affiliated Hospital of Nanjing University Medical School. All samples were obtained with informed consent from patients, according to the Declaration of Helsinki. In addition, the acquisition of lung tissue and BALF samples was approved by the Institutional Review Board of Nanjing Drum Tower Hospital.

### Mice

C57BL/6, *Nlrp3*^-/-^, *Caspase-1*^-/-^, *Gsdmd*^-/-^, *Gsdme*^-/-^ and *Gsdmd*^-/-^*Gsdme*^-/-^ mice were provided by Model Animal Research Center of Nanjing University. The mice were maintained in an SPF animal facility that is accredited by the Association for Assessment and Accreditation of Laboratory Animal Care International.

### Clinical samples

The present study was approved by the Ethics Committee of the Medical School of Nanjing University. Written informed consent was obtained from all patients enrolled in this study. Human lung tissue samples were obtained from 8 patients with silicosis (mean age±SD: 65.8±4.58 years; 1 female, 7 males) who had undergone lung transplantation surgery in the Lung Transplant Center of Wuxi People’s Hospital (Wuxi, PR China). A diagnosis of silicosis was made based on exposure and typical radiological findings, according to the ILO’s International Classification of Radiographs of Pneumoconiosis [51]. Six control lung tissues (mean age±SD: 66±9.75 years; 5 females, 1 male) were collected from patients undergoing surgery for cancer or pulmonary nodules in the Thoracic Surgery Department of Nanjing Drum Tower Hospital. Samples were stored at -80°C after collection.

BALF samples were obtained from 6 patients with silicosis (mean age±SD: 48±5.69 years; 6 males) and 5 patients (mean age±SD: 48.6±6.38 years; 2 females, 3 males) with chronic cough and normal chest high-resolution computed tomography (HRCT) as control subjects. The bronchoscope was wedged in a middle lobe or lingual bronchus, with at least 100 ml of preheated sterile saline instilled in five aliquots of 20 ml. BALF samples obtained by bronchoscopy were placed on ice and then centrifuged at 1500 rpm for 10 min. BALF supernatant was collected and frozen at -80°C. Detail information about the patients are show in S1 Table.

In the clinical sample analysis section, the human lung tissue proteins were extracted on ice by homogenizing with lysis buffer (50 mM Tris-HCl, pH = 7.4, 150 mM NaCl, 1% Nonidet P-40, 0.1 mM EDTA, 1 mM dithiothreitol, 0.4 mM phenylmethylsulfonyl fluoride, 0.1 mM Na3VO4, 0.1 mM NaF, and cocktail protein inhibitor). And the human BALF samples were concentrated by using Millipore Amicon Ultra-4 3KDa in 4°C. The protein concentrations were determined with a Bradford bioassay using a Bradford protein assay kit (Sangon).

Proteins were electrophoresed in SDS-PAGE gels by loading 48.2ug each sample (lung tissue) while 16.1ug each sample (BALF). The fractionated proteins were transferred to Hybond-P polyvinylidene difluoride membranes (Amersham Bioscience). Blots were blocked with 5% fat-free milk at room temperature for 1 h and then incubated with primary antibody overnight at 4°C. After being washed with TBST, the blots were incubated with secondary antibody for 1 h at room temperature. Proteins were visualized using enhanced chemiluminescence substrate (Tanon) and then quantified using a Tanon Chemiluminescent Imaging System.

### Animal experimental design and collection of mice bronchoalveolar lavage fluid

6~8-week-old C57BL/6, *Nlrp3*^-/-^, *Caspase-1*^-/-^, *Gsdmd*^-/-^, *Gsdme*^-/-^ and *Gsdmd*^-/-^*Gsdme*^-/-^ mice (25–30 g) were separately divided into 2 groups [PBS group (*n* = 3) and Silica group (*n* = 3)]. Mice were anesthetized with 80 mg/kg ketamine and 6 mg/kg xylazine (*i*.*p*.), then intranasally (*i*.*t*.) administered 0.1 ml of sterile PBS or 5 mg of silica particles in 0.1 ml of saline. BALF were obtained 14 days after silica or saline instillation. The trachea was cannulated after anesthetization, and BALF was obtained by injecting cold phosphate-buffered saline (PBS) three consecutive times to a final volume of 0.5 ml. The BALF was centrifuged at 1,500 rpm for 10 min, and the supernatant was used for cytokine determination and immunoblot analysis. Cells at the bottom were calculated to determine the relative cell number in the BALF. IL-1β and IL-18 were determined by ELISA (R&D Systems, USA) according to the manufacturer’s instructions.

### Lung histology

The harvested mouse lungs were fixed in 4% paraformaldehyde and then dehydrated in alcohol and n-butanol. The fixed tissue samples were then paraffin-embedded and cut into 5 μm sections.

H&E staining: Bring sections to Xylene for dewaxing and go through rehydration with alcohol before stain nuclei with haematoxylin. Then sections will be dehydrated with alcohol before stained with eosin for cytoplasm. Finally dehydration with alcohol and xylene followed by sealing with neutral resin.

Masson staining: Bring sections to Xylene for dewaxing and go through rehydration with alcohol before stain nuclei with haematoxylin. Then stain with Masson Ponceau S acidic reddening solution for 10 min before wash with 2% glacial acetic acid aqueous solution for 2 min. Fractionate sections with 1% phosphomolybdic acid solution for 5 min and then directly stain with aniline blue solution for 5 min without washing. Wash sections with 0.2% glacial acetic acid aqueous solution before dehydrate, clear and mount.

The degree of lung inflammation were scored blindly on a scale of 0–3 for determining Szapiel scores, and the degree of lung fibrosis were scored blindly on a scale of 0–8 for determining Ashcroft scores respectively. The mean score of Szapiel and Ashcroft for each mice were used for statistical analysis [52,53].

### *In vivo* caspase inhibitors treatment

The caspase inhibitors tested in this study were the pan-caspase inhibitor z-VAD-FMK, VX765 (Caspase-1 inhibitor), z-DEVD-FMK (Caspase-3 inhibitor) and z-IETD-FMK (Caspase-8 inhibitor). All inhibitors were purchased from MCE. Caspase inhibitors were dissolved in DMSO and further diluted with Hank’s balanced salt solution (HBSS). The dose of intraperitoneally injected caspase inhibitor(s) was 0.25 mg per mouse in a final volume of 100 μl administered once. Caspase inhibitor injection was carried out at Day 0, Day 5 and Day 10 (Fig 6) or at Day 3, Day 6, Day 9 and Day 12 after silica exposure (S6 Fig).

### Clodronate liposome administration

Clodronate liposome (100ul) or control PBS was intranasally treated after mice were anesthetized. BALF was collected for flow cytometry analysis of the number of alveolar macrophages.

### Plasmid and transfection

Complementary DNA (cDNA) for human *IL-1B* was amplified from reverse-transcribed cDNA from THP-1 cells. cDNA for human *CASPASE1*, *CASPASE3*, *CASPASE6*, and *CASPASE8* was kindly provided by Prof. Jiahuai Han from Xiamen University, China. All plasmids were constructed with the PCS2 vector. Vectors expressing pro-IL1B(pCS2-pro-IL1B) were transfected with plasmids expressing different activated caspase subunits separately (pCS2-Caspase 1 p10 + pCS2-Caspase 1 p20, pCS2-Caspase 3 p12 + pCS2-Caspase 3 p17, pCS2-Caspase 6 p11 + pCS2-Caspase 6 p18, pCS2-Caspase 8 p10 + pCS2-Caspase 8 p18) at the ratio of 1:1 into 293T cells by lipofectamide2000. After 18h, cells were harvested for western blot analysis.

### Flow cytometry

LPS-primed BMDMs derived from wild-type C57BL/6, *Nlrp3*^-/-^, *Caspase-1*^-/-^, *Gsdmd*^-/-^, *Gsdme*^-/-^ and *Gsdmd*^-/-^*Gsdme*^-/-^ mice were treated with 250 μg/ml silica. After 2 h, the cells were collected for PI staining (50ug/ml). Dead cell counting was performed by using a FACS Calibur flow cytometer (BD, USA). The data were analyzed using Flowjo software (Tree Star).

### Immunoblotting analysis

The total cell protein was extracted on ice using lysis buffer (50 mM Tris-HCl, pH = 7.4, 150 mM NaCl, 1% Nonidet P-40, 0.1 mM EDTA, 1 mM dithiothreitol, 0.4 mM phenylmethylsulfonyl fluoride, 0.1 mM Na3VO4, 0.1 mM NaF, and cocktail protein inhibitor). The protein concentrations were determined with a Bradford bioassay using a Bradford protein assay kit (Sangon). Protein (20 ug) samples were electrophoresed in 4% stacking and 10%/15% resolving SDS-PAGE gels, and the fractionated proteins were transferred to Hybond-P polyvinylidene difluoride membranes (Amersham Bioscience). Blots were blocked with 5% non-fat milk at room temperature for 1 h and were incubated with primary antibody overnight at 4°C. After being washed with TBST, the blots were incubated with secondary antibody for 1 h at room temperature. Proteins were visualized using enhanced chemiluminescence substrate (Tanon) and then quantified using a Tanon Chemiluminescent Imaging System.

### Cytotoxicity assay

LPS-primed BMDMs derived from wild-type C57BL/6, *Nlrp3*^-/-^, *Caspase-1*^-/-^, *Gsdmd*^-/-^, *Gsdme*^-/-^ and *Gsdmd*^-/-^*Gsdme*^-/-^ mice were treated with 250 μg/ml silica. After 2 h, the cell culture medium was collected for extracellular LDH release to evaluate cell death. Dead cells were removed from culture medium through centrifuge at 2,000rpm for 5min RT. LDH release was measured using a CytoTox 96 Non-Radioactive Cytotoxicity Assay kit (Promega) according to the manufacturer’s guidelines. The absorbance of the supernatant was examined at 490 nm. All values represent the percentage of LDH release compared with a maximum lysis control (1% Triton X-100-lysed cells).

### Cell culture and treatments

BMDMs were prepared from the tibiae and femora of 8- to 10-week-old mice. Cells were grown in a humidified incubator at 37°C and 5% CO2 in high-glucose DMEM supplemented with 10% fetal bovine serum (PAN) and penicillin/streptomycin in the presence of recombinant murine M-CSF (20 ng/ml). After 6 days of differentiation, the cells were used for the indicated *in vitro* experiments. HEK293T (obtained from ATCC) cells were maintained at 37°C in a humidified atmosphere of 5% CO2 in high-glucose DMEM supplemented with 10% fetal bovine serum (PAN) and penicillin/streptomycin.

Silica particle effects on cells were also evaluated in BMDMs. Macrophages were pretreated with caspase inhibitors 1 h before the end of the LPS (1 μg/ml) priming process, then treated with ATP (3 mM) or Silica (250 μg/ml) for 2 h. Whole cell lysates and supernatants were separately collected for western blot analysis and cytokine determination. Cellular morphology (by DIC) and membrane integrity (by fluorescence imaging) were continuously observed for a time course of up to 3h by confocal microscopy on a Leica LSM880 confocal LSM equipped with a 63× oil objective. The staining of the DNA by Propidium Iodide indicated plasma membrane leakage.

### Statistical analysis

The gray scale analysis of WB was done by Image J (version 2.0.0). GraphPad Prism software (version 8.0.1) was used to analyze and plot all data. Human data were presented as the median ± 95% confidence intervals(CI), and analyzed with Mann-Whitney U test by SPSS Statistics(version 23.0.0). Other statistical analyses were made with Student’s t-test. Other values are expressed as the mean ± SD of individual samples. *P*-values <0.05 was considered statistically significant.

## Supporting information

S1 Methods Supporting Materials and Methods. (DOCX)

S1 Table Detail information of patients. (TIF)

S1 Fig Flow cytometry measurement of infiltrating immune cells in lung of mice. **a** The relative number of macrophages, monocytes and neutrophils in lung tissue of mice that installed with PBS or silica, *n* = 3.
(TIF)

S2 Fig Inhibition of Gsdmd and Gsdme-dependent pyroptosis impairs immune cells infiltration in lung. **a,** Immunofluorescence images of neutrophils (CD11b^+^Ly6G^+^) and monocytes (CD11b^+^Ly6C^+^) in lung tissues of *WT*, *Nlrp3*^-/-^, *Caspase-1*^-/-^, *Gsdmd*^-/-^, *Gsdme*^-/-^ and *Gsdmd*^-/-^*Gsdme*^-/-^ mice 14 days after installation of PBS or silica, *n* = 3. Indicated cells were labelled with antibodies against CD11b (green), Ly6G/6C (red). DAPI (grey) localizes with the nuclei. Arrowheads indicate infiltrated immune cells. Scale bar represents 50 μm. **b,** Heat map of expression levels of proteins in Fig 1A, 1c and 1E, IL-18 release in the BALF of WT, *Nlrp3-/-*, *Caspase-1-/-*, *Gsdmd-/-*, *Gsdme-/-* and *Gsdmd-/-Gsdme-/-* mice 14 days after exposure to PBS or silica, n = 3. **d,**The Szapiel scores and the Ashcroft scores of lung tissues. Results are expressed as median ± 95% CI. **e,f,** LDH release (e) and PI positive cells (f) of WT and *Gsdmd-/-Gsdme-/-* BMDMs stimulated with silica for 1 h, 2 h, 4 h, 6 h and 8 h. **g,** Quantitative analysis of PI positive cells in f. (n = 3). Results are expressed as mean ± SD from three independent experiments. NS, not significant; *P<0.05, **P<0.01 and ***P<0.001.
(TIF)

S3 Fig Apoptosis caspases are required for silica-induced pyroptosis. **a,** Cell survival of primed *Nlrp3*^-/-^ BMDMs pretreated with inhibitor mixture as indicated and stimulated with silica. **b,** Cell survival of silica-stimulated *Nlrp3*^-/-^ macrophages pretreated with indicated caspase inhibitors. **c,** Cell survival of primed *Caspase-1*^-/-^ BMDMs pretreated with inhibitor mixture as indicated and stimulated with silica. **d,** Cell survival of silica-stimulated *Caspase-1*^-/-^ macrophages pretreated with indicated caspase inhibitors. **a-d**, The cell viability was measured through extracellular LDH release assay. **e,** BMDMs derived from *WT*, *Caspase-1*^-/-^ and *Gsdmd*^-/-^ mice on chambered coverslips were stimulated with silica and monitored for morphological changes over time by differential interference contrast (DIC) and fluorescence microscopy. Loss of membrane integrity was indicated by PI (red) staining of nuclear DNA. Scale bar represents 100 μm. z-VAD-FMK, pan-caspase inhibitor; C1i, VX765 (Caspase-1 inhibitor); C2i, z-VDVAD-FMK (Caspase-2 inhibitor); C3i, z-DEVD-FMK (Caspase-3 inhibitor); C6i, z-VEID-FMK (Caspase-6 inhibitor); C8i, z-IETD-FMK (Caspase-8 inhibitor); C9i, z-LEHD-FMK (Caspase-9 inhibitor); C10i, z-AEVD-FMK (Caspase-10 inhibitor); C12i, z-ATAD-FMK (Caspase-12 inhibitor). CIMix represents the mixture of all the indicated caspase inhibitors, while CIMix-C1i means lack of Caspase-1 specific inhibitor and the rest can be deduced by analogy. Results are expressed as mean ± SD from three independent experiments. *P<0.05, **P<0.01 and ***P<0.001.
(TIF)

S4 Fig Inhibitors of Caspase-1, Caspase-3, Caspase-6 and Caspase-8 block silica-induced pyroptosis. **Related to Fig 4. a,** Images and cell viability of *WT*, *Nlrp3*^-/-^, *Caspase-1*^-/-^, *Gsdmd*^-/-^, *Gsdme*^-/-^ and *Gsdmd*^-/-^*Gsdme*^-/-^ BMDMs pretreated with the indicated caspase inhibitors and stimulated with silica. Arrowheads indicate pyroptotic cells. The scale bar represents 100 μm. The cell viability checked by FACS with PI staining. **b,** The secretion of IL-1β and IL-18 from *WT*, *Nlrp3*^-/-^, *Caspase-1*^-/-^, *Gsdmd*^-/-^, *Gsdme*^-/-^ and *Gsdmd*^-/-^*Gsdme*^-/-^ BMDMs pretreated with the indicated caspase inhibitors and stimulated with silica. **c,** Gsdmd, Gsdme, Caspase-1, Caspase-3, Caspase-6, Caspase-8, IL-1β and IL-18 activation and release in both whole cell lysate and the supernatant of *WT*, *Gsdmd*^-/-^, *Gsdme*^-/-^, *Nlrp3*^-/-^ and *Caspase-1*^-/-^ BMDMs pretreated with caspase inhibitors as indicated and stimulated with ATP (3mM) and silica (0.25mg/ml). z-VAD-FMK, pan-caspase inhibitor; VX765, Caspase-1 inhibitor; z-DEVD-FMK, Caspase-3 inhibitor; z-VEID-FMK, Caspase-6 inhibitor; z-IETD-FMK, Caspase-8 inhibitor. The working concentration of each inhibitor was 50 μM. Results are expressed as mean ± SD from three independent experiments. NS, not significant; *P<0.05, **P<0.01 and ***P<0.001.
(TIF)

S5 Fig Silencing of Caspase-3, Caspase-6 and Caspase8 reduced silica-induced cell death. **Related to Fig 4. a,** Immunoblotting analysis of the siRNA efficacy targeting Caspase-3, Caspase-6 and Caspase-8 in *WT*, *Gsdme*^-/-^, *Caspase-1*^-/-^, *Gsdmd*^-/-^ and *Nlrp3*^-/-^ BMDMs. **b,** Images of Caspase-3, Caspase-6 or Caspase-8 downregulated-BMDMs that stimulated with silica or ATP. Arrowheads indicate pyroptotic cells. Scale bar represents 100 μm (data representative of three independent experiments). **C,** Gsdmd, Gsdme, Caspase-1, Caspase-3, Caspase-6, Caspase-8, IL-1β and IL-18 activation and release in both whole cell lysate and the supernatant of *WT*, *Gsdmd*^-/-^, *Gsdme*^-/-^, *Caspase-1*^-/-^ and *Nlrp3*^-/-^ BMDMs transfected with siRNA as indicated after silica or ATP treatment. * represents non-specific bands.
(TIF)

S6 Fig *In vivo* treatment of caspase inhibitors after silica exposure alleviates immune cells infiltration and silicosis pathology. **Related to Fig 6. a,** Caspase inhibitors treatment strategy. **b,** The levels of IL-1β and IL-18 in BALF of mice, n = 3. **c,** Relative number of macrophages, monocytes and neutrophils in lung tissue of mice that installed with PBS, silica and indicated inhibitors, *n* = 3. **d,** The number of macrophages, monocytes and neutrophils in the whole lungs of mice 14 days after instillation of PBS, silica and the indicated inhibitors, *n* = 3. **e,** Immunofluorescence images of macrophages (CD11b^+^F480^+^), neutrophils (CD11b^+^Ly6G^+^) and monocytes (CD11b^+^Ly6C^+^) in lung tissues of mice. Arrowheads indicate the infiltrated immune cells that labelled with antibodies against CD11b (green), F4/80 (red) and Ly6G/6C (red). DAPI (grey) localizes with the nuclei. The scale bar represents 50 μm. **f,** H&E (upper) and Masson (lower) staining of the indicated mouse lung sections 14 days after the initial silica challenge. The scale bar represents 100 μm, *n =* 3. **g**, The Szapiel scores of the H&E staining and the Ashcroft scores of Masson staining. Results are expressed as median ± 95% CI. z-VAD, z-VAD-FMK, pan-caspase inhibitor; 3is, VX765+z-DEVD-FMK+z-IETD-FMK. VX765, Caspase-1 inhibitor; z-DEVD-FMK, Caspase-3 inhibitor; z-IETD-FMK, Caspase-8 inhibitor. The total dosage of injected caspase inhibitor(s) was 0.25 mg per mouse administered once. a-f Results are expressed as mean ± SD from three independent experiments. NS, not significant; *P<0.05, **P<0.01 and ***P<0.001.
(TIF)

S7 Fig Inhibition of Gsdmd and Gsdme by dimethyl fumarate blocks silica-induced pyroptosis. **Related to Fig 6. a,** Images of *WT* and *Gsdmd*^-/-^ macrophages pretreated with DMF (0, 25, 50 and 100 μM) and stimulated with silica for 2 h. Arrowheads (red) indicate pyroptotic cells. The scale bar represents 100 μm. **b,** Cell death of *WT* and *Gsdmd*^-/-^ macrophages were measured via LDH assay. Results are expressed as mean ± SD from three independent experiments. **c,** The Szapiel scores of the H&E staining and the Ashcroft scores of Masson staining. Results are expressed as median ± 95% CI. NS, not significant; *P<0.05, **P<0.01 and ***P<0.001.
(TIF)
