# Supplementary material for: Blocking Caspase-1/Gsdmd and Caspase-3/-8/Gsdme pyroptotic pathways rescues silicosis in mice
Source: PLoS Genet. 2022 Dec 2;18(12):e1010515. doi: 10.1371/journal.pgen.1010515 (PMC9718385; doi:10.1371/journal.pgen.1010515)
Supplement: S1 Methods — (DOCX) [file pgen.1010515.s001.docx]

Supplementary Materials for

Blocking Caspase-1/Gsdmd and Caspase-3/-8/Gsdme pyroptotic pathways rescues silicosis in mice

Lulu Kang, Jinghong Dai, Yufang Wang, Peiliang Shi, Yujie Zou, Jingwen Pei, Yaqiong Tian, Ji Zhang, Visarut Codey Buranasudja, Jingyu Chen, Hourong Cai, Xiang Gao, Zhaoyu Lin

Correspondence to: Jingyu Chen ([chenjy@wuxiph.com](mailto:chenjy@wuxiph.com)); Hourong Cai (caihourong2013@163.com); Xiang Gao (gaoxiang@nju.edu.cn) and Zhaoyu Lin (linzy@nju.edu.cn)

**Materials and Methods**

**Flow Cytometry Analysis of immune cell infiltration in lung tissue**

The digestion solution used to digest lung tissues was composed of 10mM HEPES, 10ug/ml DNAse I, Collagenase D (0.4mg/mL), 2% fatal bovine serum and PBS containing Ca2+ and Mg2+. The lung tissues were cut into small pieces, digested in a 37°C water bath for 30 minutes and mix well. After the digestion process is completed, grind digested tissues with a 70uM filter. Then wash the cells, centrifuge, and discard the supernatant. If there are red blood cells, split red briefly is needed; if there are no red blood cells, wash the cells with PBS. Then incubate the antibody at 4°C for 30-60min protecting from light, and wash twice before the test. Analysis of infiltrating cells was performed by using a BD LSRFortessa flow cytometer (BD, USA). The data were analyzed using Flowjo software (Tree Star).

**Immunofluorescence of Lung Sections**

Fresh lung tissues were fixed with 4% Paraformaldehyde overnight and dehydrated with 30% sucrose for 24 hours. Then tissues were embedded with optimal cutting temperature compound (OCT) and cut with Leica Biosystems Cryostats. Frozen sections of lung tissue were washed with PBS for several minutes. Then, the sections were permeabilized with 0.1% Triton X-100 for 30 min and blocked with 5% BSA at RT for 30 min. Then, the sections were incubated with primary antibody diluted in blocking buffer at 4°C overnight. On the following day, the sections were washed in PBS and incubated with secondary antibody at RT for 1 h. After washing with PBS, the sections were mounted with anti-fade mounting media (VECTASHIELD, H-1000) and photographed using a confocal microscope (Zeiss LSM880).

**Antibodies and reagents**

Antibodies against human GSDMD (ab209845), GSDME/DFNA5 (ab215191) and human CASP1 (ab207802) were purchased from Abcam (UK). The antibody against IL-1β (5129) and IL-18 (5810R) were purchased from BioVision (CA, USA). The antibody against mouse Caspase-1 (5B10) was purchased from eBioscience (CA, USA). The antibodies against Caspase-3 (#9665S), Caspase-6 (#9762), HA (#3724) and Flag (#8146) were obtained from Cell Signaling Technology (MA, USA). The antibody against Caspase-8 (1G12) was purchased from Enzo Life Sciences (NY, USA). The antibodies against Ly6G/6C (RB6-8C5), F4/80 (CI-A3-1) and CD11b were obtained from Novus (NY, USA). The GFP (B-2) antibody was obtained from Santa Cruz (TX, USA). Propidium iodide and ATP were purchased from Sangon Biotech (China). Silica crystals (MIN- U-SIL 15) were obtained from U.S. Silica (MD, USA). Lipopolysaccharide, FITC-conjugated goat antibody against rabbit IgG (F9887) and α-tubulin antibody (T5168) were purchased from Sigma-Aldrich (MO, USA). Cy5-conjugated goat antibody against rat IgG (112-175-003) was purchased from Jackson ImmunoResearch (PA, USA). The caspase screening reagents used in Fig 4 and Fig S3 were purchased from R&D Systems (MN, USA). The z-VAD-FMK, VX765, z-DEVD-FMK and z-IETD-FMK used in Fig 5 and figs. S4, 5, 6 and 7 were obtained from MCE (NJ, USA). z-VEID-FMK was purchased from APExBIO (TX, USA). Nec-1 and NSA were purchased from MCE (NJ, USA). siRNA smart pools targeting Caspase-3, Caspase-6 and Caspase-8 were purchased from GE Healthcare (UK). Lipofectamine RNAiMax was purchased from Life Technologies (CA, USA). M-CSF was purchased from Peprotech (NJ, USA).

**Gene knockdown of caspases**

siRNA smart pools that targeted Caspase-3, Caspase-6 and Caspase-8 were transfected with Lipofectamine RNAiMax, after 72 h, primed cells were stimulated with silica and ATP. The working concentration of the transfected siRNA pool was 100 nM.

**Plasmid cloning**

cDNAs for human GSDME and NLRP3 were provided by Jiahuai Han (Xiamen University). cDNAs for ASC, IL1B, CASP3, CASP6 and CASP8 subunits as well as proCASP1 and proCASP8 were amplified from reverse-transcribed cDNA of THP-1 cells. The cDNAs for CASP1, CASP3, CASP6 and CASP8 subunits as well as proCASP1 and proCASP8 were inserted into the pcDNA5-FRT-HA vector for transient expression in 293T cells. ASC cDNA was inserted into the pEGFP-N1 vector. NLRP3, IL1B and GSDME cDNAs were inserted into the pcDNA5-FRT-Flag vector. All plasmids were verified by DNA sequencing.
