# Supplementary figures and images for: Blocking Caspase-1/Gsdmd and Caspase-3/-8/Gsdme pyroptotic pathways rescues silicosis in mice
Source: PLoS Genet. 2022 Dec 2;18(12):e1010515. doi: 10.1371/journal.pgen.1010515 (PMC9718385; doi:10.1371/journal.pgen.1010515)

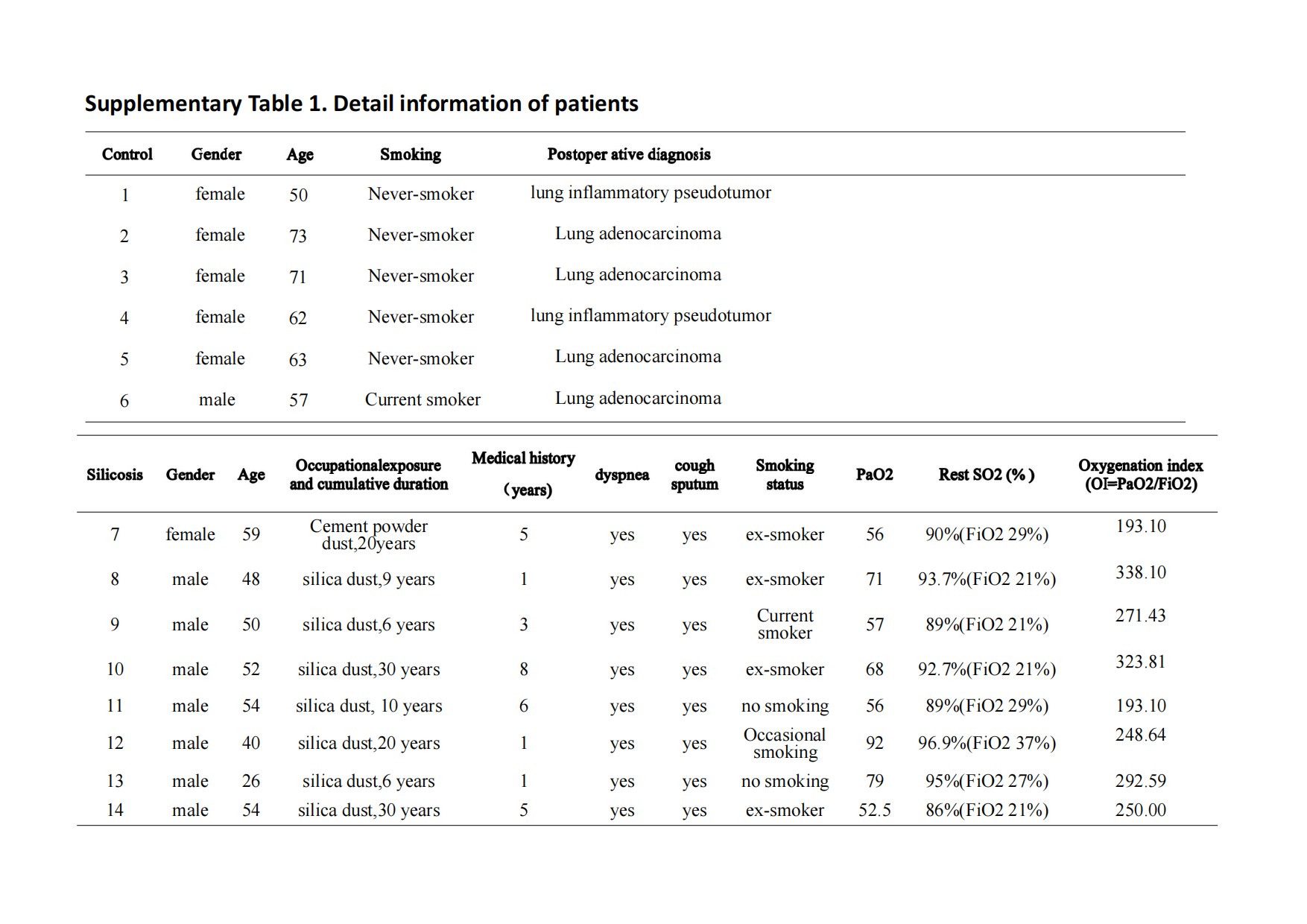

Supplement: S1 Table — (TIF) [file pgen.1010515.s002.tif]

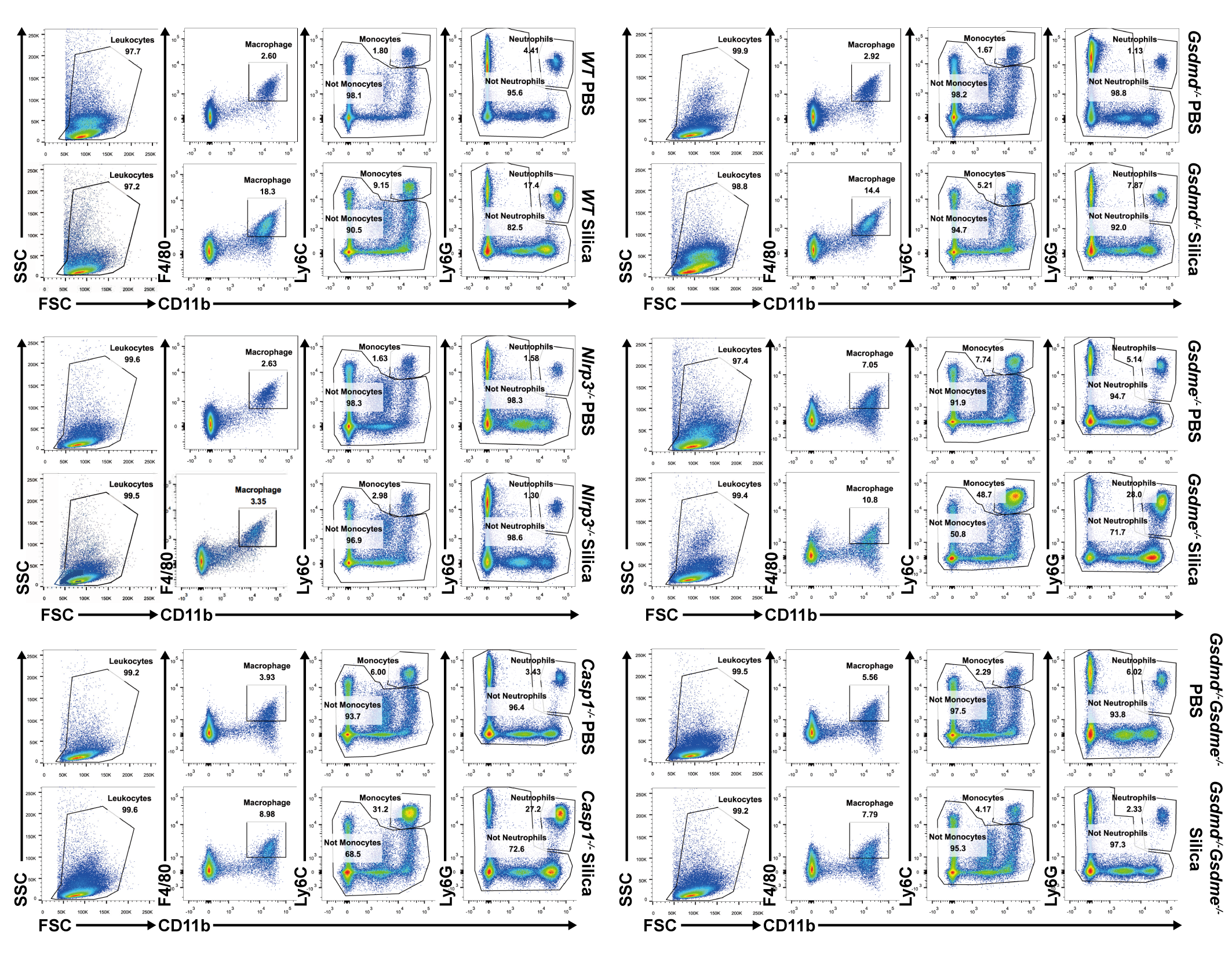

Supplement: S1 Fig — a The relative number of macrophages, monocytes and neutrophils in lung tissue of mice that installed with PBS or silica, n = 3. (TIF) [file pgen.1010515.s003.tif]

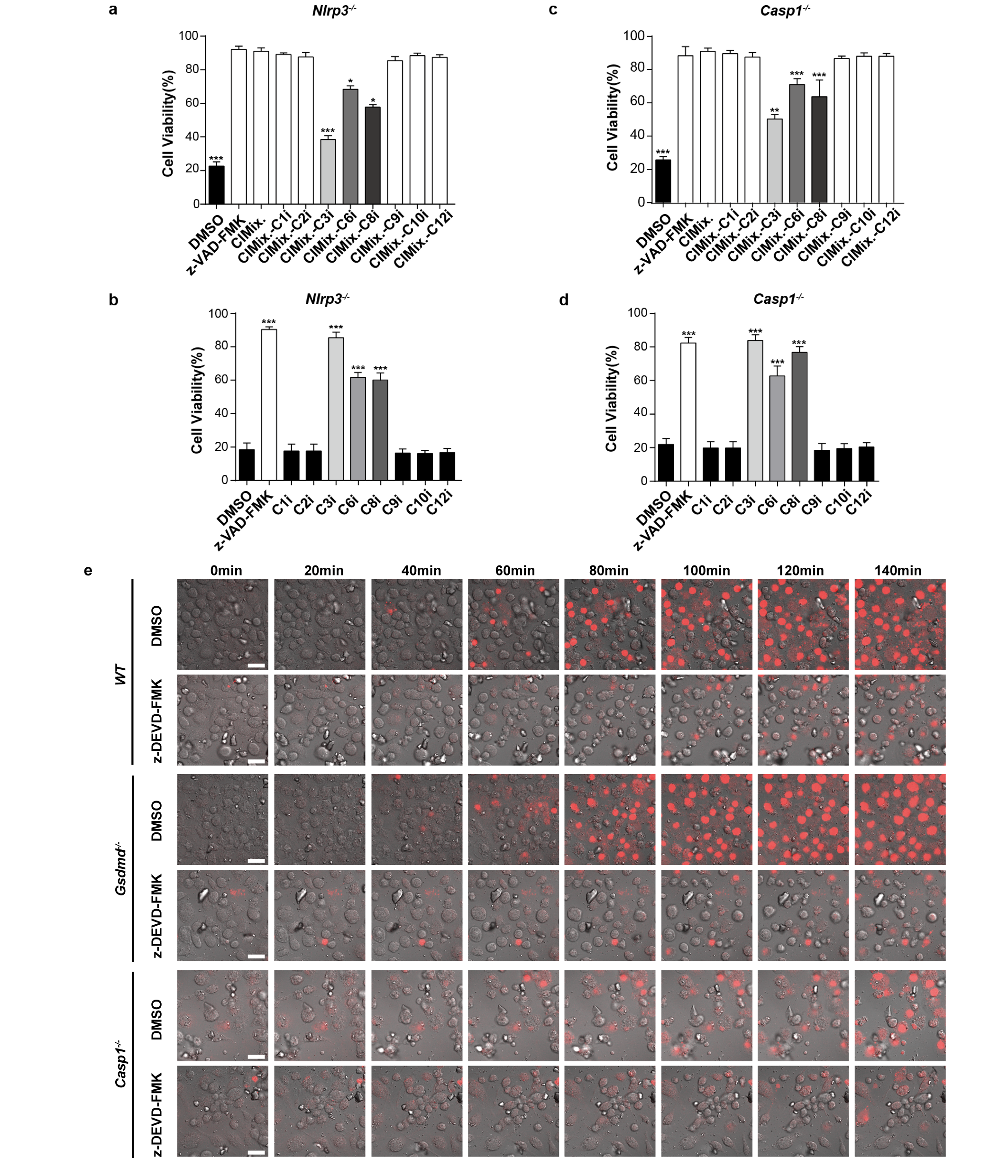

Supplement: S3 Fig — a, Cell survival of primed Nlrp3-/- BMDMs pretreated with inhibitor mixture as indicated and stimulated with silica. b, Cell survival of silica-stimulated Nlrp3-/- macrophages pretreated with indicated caspase inhibitors. c, Cell survival of primed Caspase-1-/- BMDMs pretreated with inhibitor mixture as indicated and stimulated with silica. d, Cell survival of silica-stimulated Caspase-1-/- macrophages pretreated with indicated caspase inhibitors. a-d, The cell viability was measured through extracellular LDH release assay. e, BMDMs derived from WT, Caspase-1-/- and Gsdmd-/- mice on chambered coverslips were stimulated with silica and monitored for morphological changes over time by differential interference contrast (DIC) and fluorescence microscopy. Loss of membrane integrity was indicated by PI (red) staining of nuclear DNA. Scale bar represents 100 μm. z-VAD-FMK, pan-caspase inhibitor; C1i, VX765 (Caspase-1 inhibitor); C2i, z-VDVAD-FMK (Caspase-2 inhibitor); C3i, z-DEVD-FMK (Caspase-3 inhibitor); C6i, z-VEID-FMK (Caspase-6 inhibitor); C8i, z-IETD-FMK (Caspase-8 inhibitor); C9i, z-LEHD-FMK (Caspase-9 inhibitor); C10i, z-AEVD-FMK (Caspase-10 inhibitor); C12i, z-ATAD-FMK (Caspase-12 inhibitor). CIMix represents the mixture of all the indicated caspase inhibitors, while CIMix-C1i means lack of Caspase-1 specific inhibitor and the rest can be deduced by analogy. Results are expressed as mean ± SD from three independent experiments. *P<0.05, **P<0.01 and ***P<0.001. (TIF) [file pgen.1010515.s005.tif]

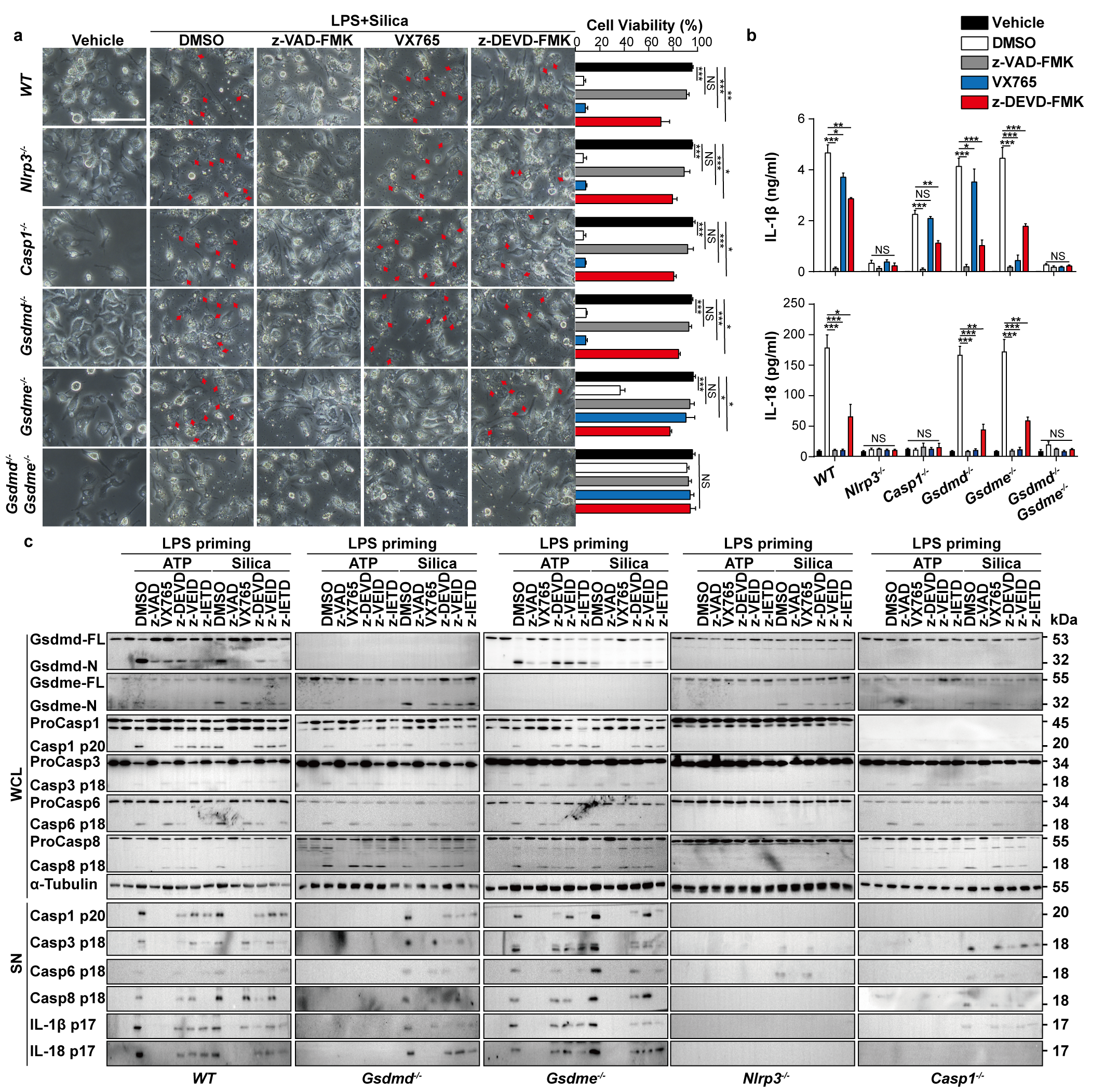

Supplement: S4 Fig — Related to Fig 4. a, Images and cell viability of WT, Nlrp3-/-, Caspase-1-/-, Gsdmd-/-, Gsdme-/- and Gsdmd-/-Gsdme-/- BMDMs pretreated with the indicated caspase inhibitors and stimulated with silica. Arrowheads indicate pyroptotic cells. The scale bar represents 100 μm. The cell viability checked by FACS with PI staining. b, The secretion of IL-1β and IL-18 from WT, Nlrp3-/-, Caspase-1-/-, Gsdmd-/-, Gsdme-/- and Gsdmd-/-Gsdme-/- BMDMs pretreated with the indicated caspase inhibitors and stimulated with silica. c, Gsdmd, Gsdme, Caspase-1, Caspase-3, Caspase-6, Caspase-8, IL-1β and IL-18 activation and release in both whole cell lysate and the supernatant of WT, Gsdmd-/-, Gsdme-/-, Nlrp3-/- and Caspase-1-/- BMDMs pretreated with caspase inhibitors as indicated and stimulated with ATP (3mM) and silica (0.25mg/ml). z-VAD-FMK, pan-caspase inhibitor; VX765, Caspase-1 inhibitor; z-DEVD-FMK, Caspase-3 inhibitor; z-VEID-FMK, Caspase-6 inhibitor; z-IETD-FMK, Caspase-8 inhibitor. The working concentration of each inhibitor was 50 μM. Results are expressed as mean ± SD from three independent experiments. NS, not significant; *P<0.05, **P<0.01 and ***P<0.001. (TIF) [file pgen.1010515.s006.tif]

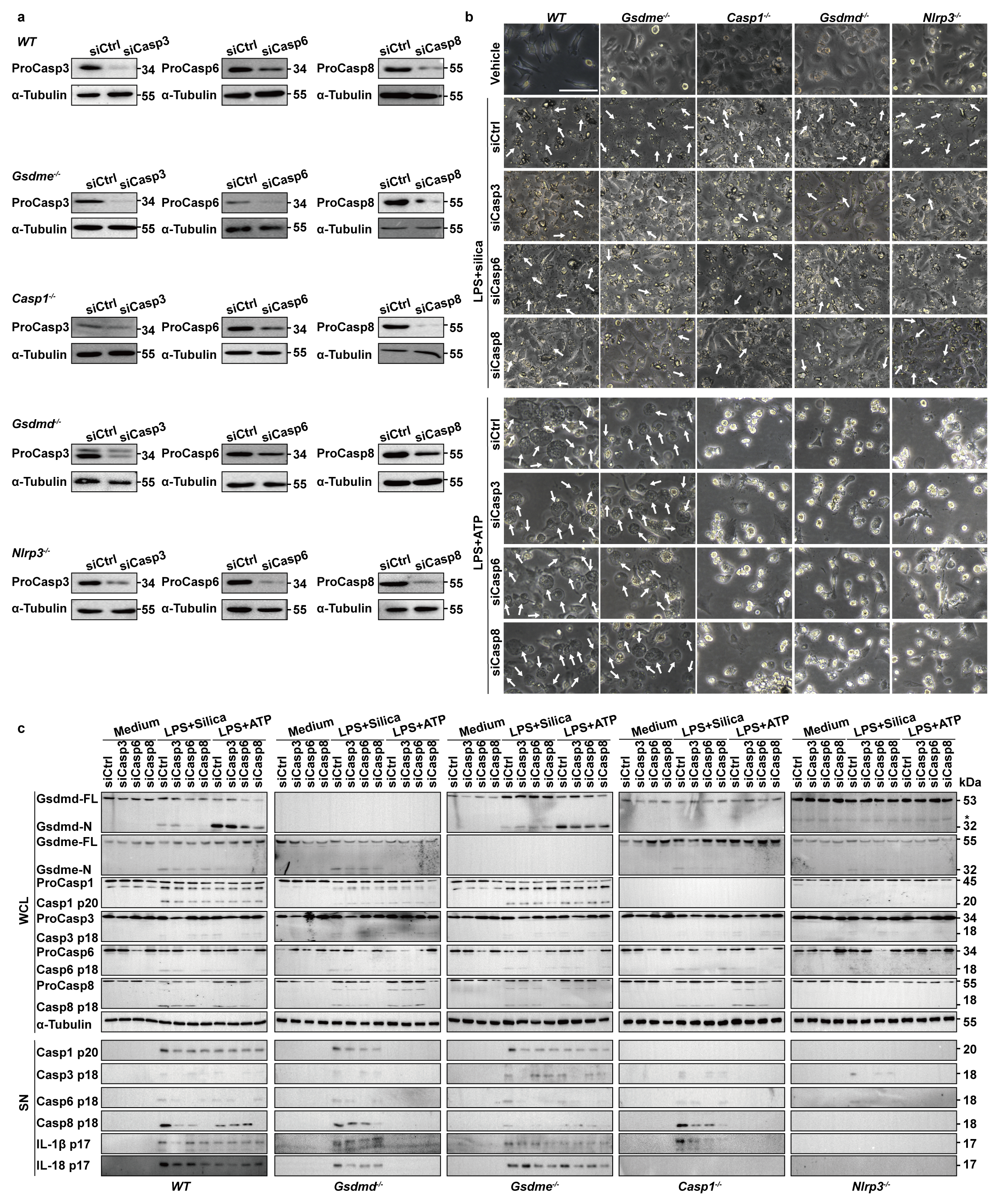

Supplement: S5 Fig — Related to Fig 4. a, Immunoblotting analysis of the siRNA efficacy targeting Caspase-3, Caspase-6 and Caspase-8 in WT, Gsdme-/-, Caspase-1-/-, Gsdmd-/- and Nlrp3-/- BMDMs. b, Images of Caspase-3, Caspase-6 or Caspase-8 downregulated-BMDMs that stimulated with silica or ATP. Arrowheads indicate pyroptotic cells. Scale bar represents 100 μm (data representative of three independent experiments). C, Gsdmd, Gsdme, Caspase-1, Caspase-3, Caspase-6, Caspase-8, IL-1β and IL-18 activation and release in both whole cell lysate and the supernatant of WT, Gsdmd-/-, Gsdme-/-, Caspase-1-/- and Nlrp3-/- BMDMs transfected with siRNA as indicated after silica or ATP treatment. * represents non-specific bands. (TIF) [file pgen.1010515.s007.tif]

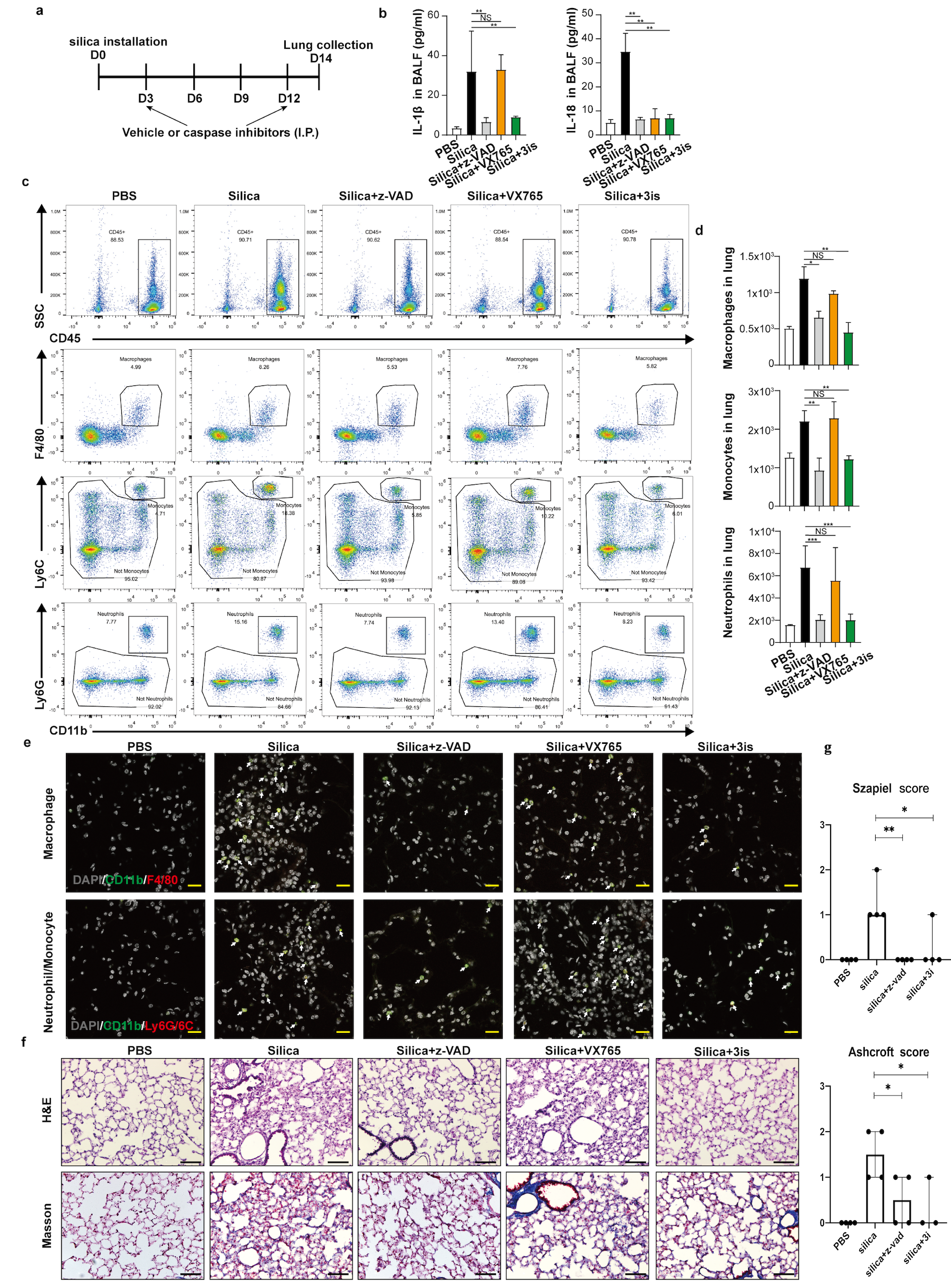

Supplement: S6 Fig — Related to Fig 6. a, Caspase inhibitors treatment strategy. b, The levels of IL-1β and IL-18 in BALF of mice, n = 3. c, Relative number of macrophages, monocytes and neutrophils in lung tissue of mice that installed with PBS, silica and indicated inhibitors, n = 3. d, The number of macrophages, monocytes and neutrophils in the whole lungs of mice 14 days after instillation of PBS, silica and the indicated inhibitors, n = 3. e, Immunofluorescence images of macrophages (CD11b+F480+), neutrophils (CD11b+Ly6G+) and monocytes (CD11b+Ly6C+) in lung tissues of mice. Arrowheads indicate the infiltrated immune cells that labelled with antibodies against CD11b (green), F4/80 (red) and Ly6G/6C (red). DAPI (grey) localizes with the nuclei. The scale bar represents 50 μm. f, H&E (upper) and Masson (lower) staining of the indicated mouse lung sections 14 days after the initial silica challenge. The scale bar represents 100 μm, n = 3. g, The Szapiel scores of the H&E staining and the Ashcroft scores of Masson staining. Results are expressed as median ± 95% CI. z-VAD, z-VAD-FMK, pan-caspase inhibitor; 3is, VX765+z-DEVD-FMK+z-IETD-FMK. VX765, Caspase-1 inhibitor; z-DEVD-FMK, Caspase-3 inhibitor; z-IETD-FMK, Caspase-8 inhibitor. The total dosage of injected caspase inhibitor(s) was 0.25 mg per mouse administered once. a-f Results are expressed as mean ± SD from three independent experiments. NS, not significant; *P<0.05, **P<0.01 and ***P<0.001. (TIF) [file pgen.1010515.s008.tif]

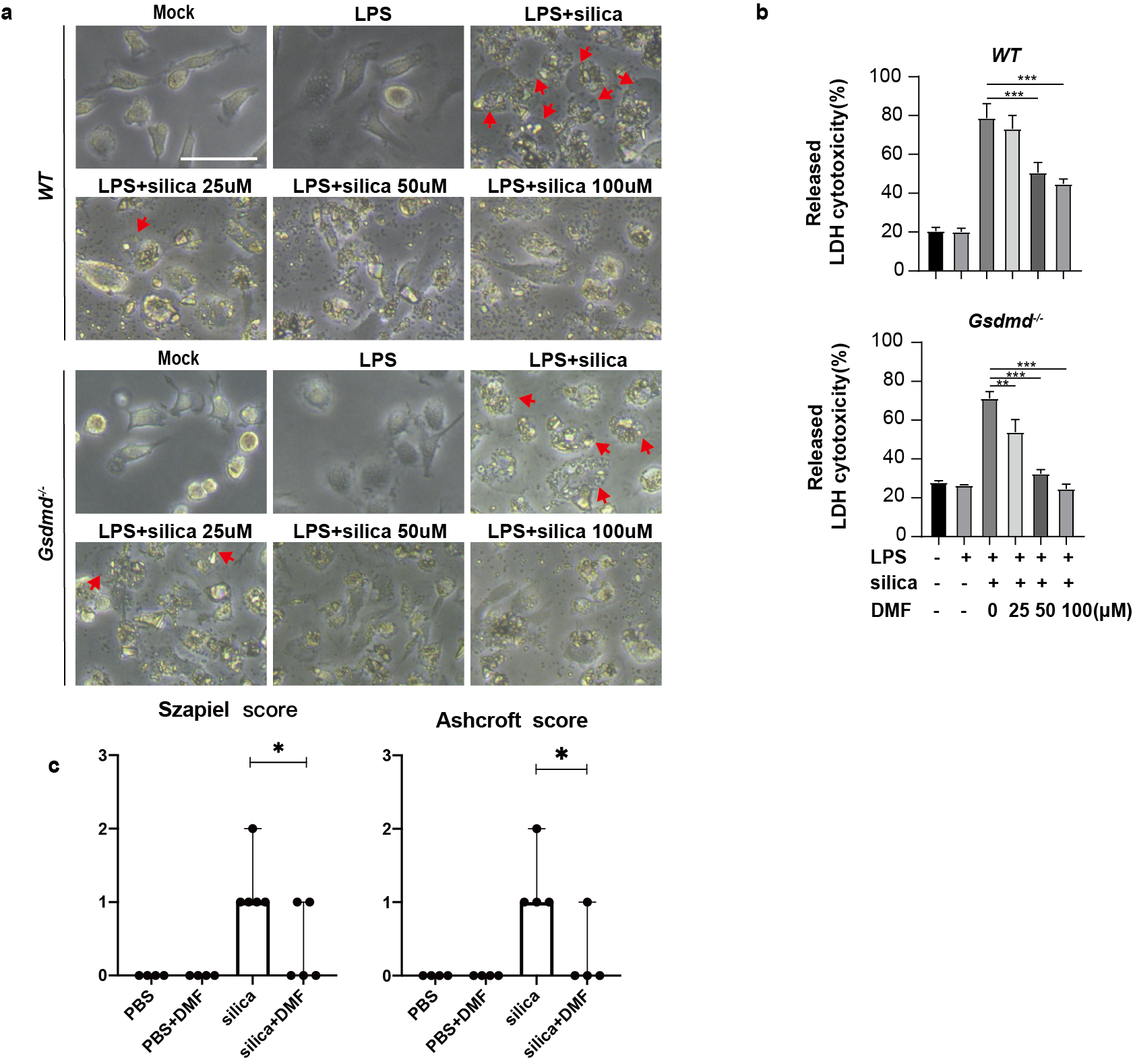

Supplement: S7 Fig — Related to Fig 6. a, Images of WT and Gsdmd-/- macrophages pretreated with DMF (0, 25, 50 and 100 μM) and stimulated with silica for 2 h. Arrowheads (red) indicate pyroptotic cells. The scale bar represents 100 μm. b, Cell death of WT and Gsdmd-/- macrophages were measured via LDH assay. Results are expressed as mean ± SD from three independent experiments. c, The Szapiel scores of the H&E staining and the Ashcroft scores of Masson staining. Results are expressed as median ± 95% CI. NS, not significant; *P<0.05, **P<0.01 and ***P<0.001. (TIF) [file pgen.1010515.s009.tif]
